# Multiple-Allele MHC Class II Epitope Engineering by a Molecular Dynamics-Based Evolution Protocol

**DOI:** 10.3389/fimmu.2022.862851

**Published:** 2022-04-27

**Authors:** Rodrigo Ochoa, Victoria Alves Santos Lunardelli, Daniela Santoro Rosa, Alessandro Laio, Pilar Cossio

**Affiliations:** ^1^ Biophysics of Tropical Diseases, Max Planck Tandem Group, University of Antioquia UdeA, Medellin, Colombia; ^2^ Department of Microbiology, Immunology and Parasitology, Federal University of Sao Paulo, Sao Paulo, Brazil; ^3^ Institute for Investigation in Immunology (iii), Instituto Nacional de Ciência e Tecnologia (INCT), Sao Paulo, Brazil; ^4^ Physics Area, International School for Advanced Studies (SISSA), Trieste, Italy; ^5^ Condensed Matter and Statistical Physics Section, International Centre for Theoretical Physics (ICTP), Trieste, Italy; ^6^ Department of Theoretical Biophysics, Max Planck Institute of Biophysics, Frankfurt am Main, Germany; ^7^ Center for Computational Mathematics, Flatiron Institute, New York, NY, United States; ^8^ Center for Computational Biology, Flatiron Institute, New York, NY, United States

**Keywords:** MHC class II, epitope engineering, molecular dynamics, peptide design, multiple-allele binding

## Abstract

Epitopes that bind simultaneously to all human alleles of Major Histocompatibility Complex class II (MHC II) are considered one of the key factors for the development of improved vaccines and cancer immunotherapies. To engineer MHC II multiple-allele binders, we developed a protocol called PanMHC-PARCE, based on the unsupervised optimization of the epitope sequence by single-point mutations, parallel explicit-solvent molecular dynamics simulations and scoring of the MHC II-epitope complexes. The key idea is accepting mutations that not only improve the affinity but also reduce the affinity gap between the alleles. We applied this methodology to enhance a *Plasmodium vivax* epitope for multiple-allele binding. *In vitro* rate-binding assays showed that four engineered peptides were able to bind with improved affinity toward multiple human MHC II alleles. Moreover, we demonstrated that mice immunized with the peptides exhibited interferon-gamma cellular immune response. Overall, the method enables the engineering of peptides with improved binding properties that can be used for the generation of new immunotherapies.

## 1 Introduction

**Figure 1:**
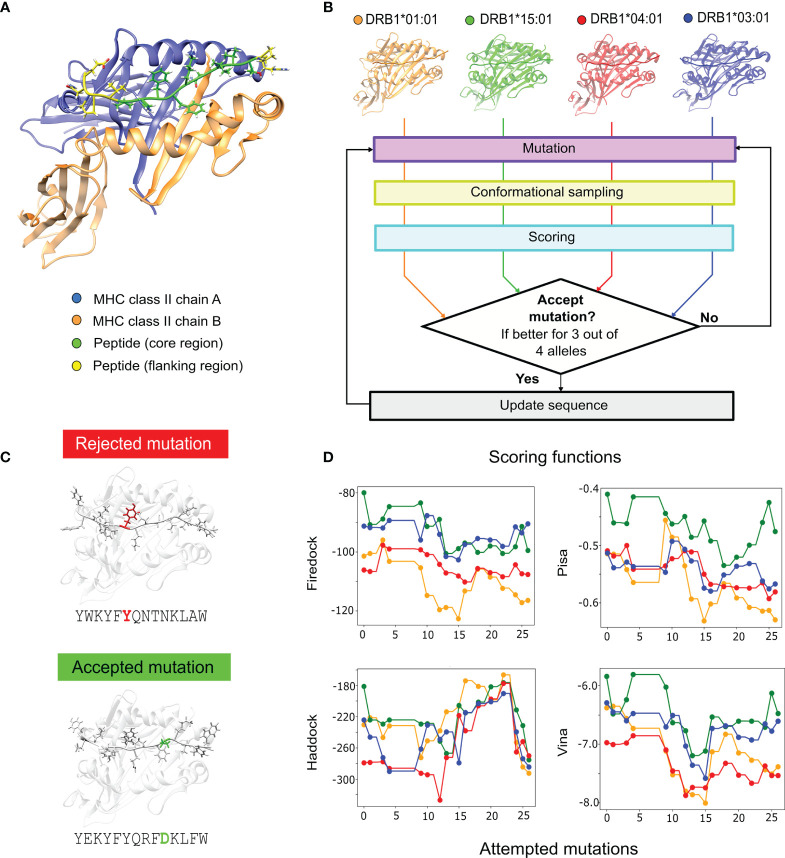
Multiple-allele epitope engineering protocol. **(A)** MHC II receptor bound to a peptide, with chain α represented in blue, chain β in orange, the peptide core region in green and the peptide flanking regions in yellow. **(B)** PanMHC-PARCE workflow for epitope optimization toward multiple alleles (DRB1*01:01 in orange, DRB1*15:01 in green, DRB1*04:01 in red and DRB1*03:01 in blue). First, the starting protein-peptide complexes are modelled. Then, a Monte Carlo algorithm is performed. At each step, a single-point mutation on the peptide is attempted. Then, parallel simulations of the mutated peptide with each allele are sampled with MD. The trajectory snapshots, for each allele, are scored with multiple scoring functions, and a consensus criterion is applied. The mutation is accepted if the scoring consensus is favorable for three out of the four alleles. **(C)** An example of a rejected mutation during the design is colored in red, and an accepted mutation colored in green. **(D)** Example of the evolution of the scoring results for the multiple-allele engineering of the *P. vivax* epitope for Firedock, Pisa, Haddock and Vina (see the Methods). The dots in the curves represent the mutations that are accepted and the colors of the different MHC alleles are based on panel **(B)**.

Peptides have been used in vaccine formulations to trigger specific immune responses toward a particular disease (1). These peptides, acting as epitopes, are able to bind receptors such as the Major Histocompatibility Complex class II (MHC II) (e.g., **Figure 1A**), which displays them on cell surfaces for T-cell recognition and immune-response activation (2). The use of artificial peptides capable of mimicking natural epitopes has also been proposed (3). For example, designed peptides have been used for immunological therapies (4), and as neoepitopes targeting tumor-specific mutations, with the potential for becoming cancer vaccines (5, 6).

Peptides have also been successfully used as adjuvants, namely components of a vaccine which increase the immune response (7–9). A primary example of this is PADRE (Pan DR T-helper Epitope), which was developed as a universal T-helper epitope (10). This peptide helps to trigger the immunological machinery, and complements the specific response generated by the antigenic sub-units (11). PADRE was designed through multiple and expensive experimental trials. The targets of PADRE, and of many other adjuvant peptides, are MHC II receptors.

In humans, MHC II exists in several different alleles, some of which involve mutations localized in the peptide-binding pocket. Consequently, peptides targeting this receptor typically have different binding affinities for different alleles. Multiple-allele T-helpers could be incorporated into vaccines and immunological agents, leading to an improved activity for all human populations (2, 12). For example, PADRE binds with high affinity to multiple alleles, and has been evaluated *in vitro* and *in vivo* with positive results (13, 14). However, PADRE has no pathogen specificity (*i.e.*, no information about pathogenic epitopes was used to create its consensus sequence), which limits its capability to target a specific disease. Moreover, designing peptides through a trial-and-error experiments is extremely challenging, given the large amount of possible mutations, and the costs and time required to test potential candidates experimentally (15).

Machine learning models trained on peptide-sequence datasets (16), aid the prediction of binding affinities of epitopes toward different MHC II alleles (17, 18). The IEDB consensus tool (19) combines three methods [CombLib (20), SMM-align (21) and Sturniolo (22)] to score epitopes by comparing against five million random peptide-sequences toward specific MHC II alleles. NetMHCIIPan (23) predicts peptide-binding to any MHC II molecule using artificial neural networks. Machine learning strategies can predict the likelihood of antigen presentation in the context of specific HLA class I (24) or for both class I and II alleles, trained with metadata such as mass spectroscopy (25, 26). Multiple-allele scoring prediction of MHC II epitopes has also been developed using machine learning methods trained with a variety of data (27, 28). However, the extension of these methods to engineer new epitope sequences with improved binding is challenging because large affinity changes can be triggered by single-point mutations, which are difficult to predict (29).

To address this problem, one can resort to *de novo* design, in which peptides are engineered based on the physico-chemical properties of their interaction with the targets. This route has been successful in the design of antimicrobial and membrane pore-forming peptides (30, 31), or peptides capable of binding with high affinity to organic molecules (32), and of antibody fragments (33). The wide availability of MHC I and II structures (34, 35) has greatly facilitated targeted peptide-binding design. Recently, it has been shown that structural modelling toward MHC I, in combination with NetMHC predictions, brings valuable insights of neoepitopes’ immunogenicity (36). *De novo* design of MHC I binders targeting multiple alleles has also been attempted (37). Rosetta applications (38) have been used to design epitope scaffolds for neutralizing antibodies (39), and to reduce the immunogenicty of a target protein by searching for its potential epitopes and designing less immunogenic sequences (40). Recently, an energy term for the same software has been customized to *deimmunize* biological entities toward MHC II (41). However, engineering peptides to have higher affinities toward multiple MHC II alleles is still a major challenge, due to its highly dynamic nature.

In this work, we developed an *in silico* design approach to address these challenges, and engineer peptides with improved *experimental* binding affinity toward many MHC II alleles simultaneously. Based on extensive molecular dynamics (MD) simulations, our novel methodology runs multiple designs (42) in parallel, one for each allele, and automatically selects epitope mutations which make the affinity higher for the majority of the alleles, rejecting those which increase the affinity unevenly. Using this approach, we engineered modifications to a *Plasmodium vivax* (*P. vivax*) epitope to improve simultaneously its affinity toward several human MHC II (HLA) alleles. A small subset of sequences was tested *in vitro*, finding four modified peptides with better affinities. To confirm their ability to induce specific immune responses *ex vivo*, each peptide was mixed with the adjuvant AddaVax and used to immunize C57BL/6 mice. All peptides induced specific in1terferon gamma (IFNγ)-producing cells and, the engineered peptides remained immunogenic. This work will open the route to *in silico* multiple-allele epitope engineering.

## 2 Results

### 2.1 Multiple-Allele Peptide Engineering Protocol

The multiple-allele engineering protocol enables the design of peptides capable of binding with high affinity to multiple targets (**Figure 1**). Its key innovation is to run in parallel multiple PARCE (42) simulations, accepting mutations that increase the affinity to the majority of targets. The protocol starts with a given epitope sequence, and requires building an atomistic model of the peptide bound to each allele. **Figure 1A** shows an example of a starting structure for a single MHC II allele. A sequence of single-point mutations is then attempted (see the Methods). MD simulations are ran in parallel, one for each allele with the new mutated peptide (**Figure 1B**). Each MD trajectory is then scored using several scoring functions (see the Methods). The mutation is considered favorable for each allele if the majority of scoring functions provide a positive score. Then, the new peptide sequence is accepted, if it is favorable for at least 75% of the alleles; otherwise, the sequence is rejected (**Figure 1C**). Therefore, the protocol is accepting mutations that not only improve the affinity but importantly reduce the affinity gap between the alleles. The process is iterated until the estimated affinity toward the target does not significantly improve. **Figure 1D** shows an example of the evolution of the scoring functions, which collectively shift toward better scores for the multiple alleles. An animation of the design process is shown in **Supplementary Video 1** for an Influenza A virus peptide.

### 2.2 Optimization Design of an Influenza Epitope Toward a Single MHC II Allele

**Figure 2:**
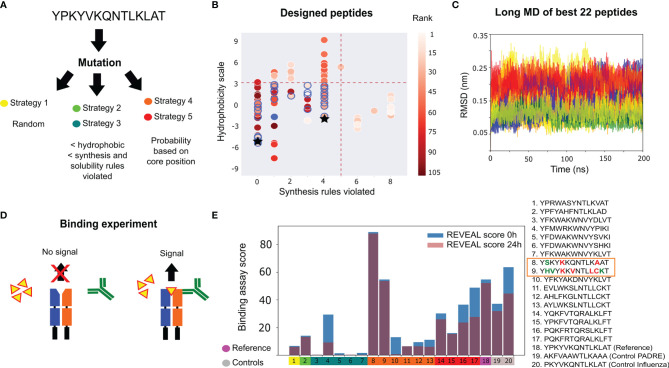
Influenza peptide design for binding to a single allele. **(A)** Design strategies implemented: random mutations (strategy 1), mutations filtered by bioinformatic properties (strategies 2 and 3) and mutations filtered by MHC II binding motifs (strategies 4 and 5). **(B)** Hydrophobicity values, the number of synthesis rules violated and the average rank (scoring from 5 ns MD simulations) for the designed peptides. The thresholds (red dashed lines) used for selecting 22 peptides (blue circles) for long MD simulations. The original peptide is represented by a blue-filled circle. The controls are represented as black stars. **(C)** Cα RMSD to the starting structure of the 22 selected peptides during the long MD, colored by the mutation strategy panel **(A)**. **(D)** Illustration of the Proimmune REVEAL binding assay. The peptide (yellow) binds the MHC II protein. When the components bind in the correct conformation, a positive signal is generated *via* a labelled antibody (green). **(E)** Experimental results of the REVEAL assay for the 17 designed peptides, the original sequence and two positive controls (PADRE and Influenza peptide PKYVKQNTLKLAT). The REVEAL binding score is a value between 0 and 100, which is determined by the comparison with a control T-cell epitope. The peptides are split into the five mutation strategies [color code as in panel **(A)**]. The scores are shown at 0 hours (blue bar) and after 24 hours (purple bar). The box highlights the two peptides with better binding than the reference sequence, with the modifications in bold, and colored in red and green to represent changes in the core and flanking regions, respectively.

We first benchmarked the protocol on the design of an epitope for a single allele of MHC II. As a starting sequence we have chosen an Influenza A virus peptide from the Hemagglutinin antigen, with sequence YPKYVKQNTLKLAT. For this epitope, structural information and binding data toward the allele DRB1*01:01 (*IC*_50_= 130 nM) are available (20). We tested different mutation strategies (**Figure 2A** and **Supplementary Note 1**) with 100 attempted mutations per strategy, in order to observe convergence of the scoring functions (see **Supplementary Figure 1**). Uniformly-distributed random mutations in the peptide sequence favour sequences with many hydrophobic amino acids, which are prone to aggregation. This motivated us to exclude sequences with many hydrophobic amino acids or those that violated many empirical peptide-synthesis rules (see **Supplementary Note 2**). We also explored the possibility of enhancing the mutation probability in the peptide core region using bioinformatics derived information (**Figure 2A**).

From the different runs, a total of 105 peptide candidates that are potentially better binders than the original peptide (see **Supplementary Tables 1–5**) were obtained. In **Figure 2B**, the average rank of each peptide is shown (using the six scoring functions and calculated over 5 ns of MD) as a function of the number of violated empirical synthesis rules and a hydrophobicity scale. We used the average rank, physicochemical and similarity filters (see Methods and **Supplementary Note 2**), to select a small set of 22 candidates for longer (200 ns) MD simulations. By monitoring several structural observables (*e.g.*, the C_α_ RMSD shown in **Figure 2C**), we selected 17 complexes that were more stable. The average rank, calculated using the average scores over the last 100 ns, was also used to select the peptide candidates for experimental testing. Two controls reporting positive binding data toward the MHC II allele (the PADRE epitope AKFVAAWTLKAAA, and an influenza immunogenic epitope PKYVKQNTLKLAT) were also scored with the same procedure (see **Supplementary Table 6** for details).

The selected candidates, the original peptide, and the two controls were synthesized and analysed using the ProImmune REVEAL^®^ MHC-peptide binding assay. This experiment assessed the level of binding to the MHC II (HLA) allele DRB1*01:01 (**Figure 2D**). The ProImmune REVEAL^®^ binding score for each MHC-peptide complex is calculated at 0 and 24 hours in comparison to the binding of the positive controls (PADRE and Influenza peptide) at 0 hours. The results are shown in **Figure 2E**. Interestingly, we found that one peptide (YSKYKKQNTLKAAT -pep8) reported a performance much superior to the original peptide sequence, and even to the PADRE control used in the experiment. This peptide only reports three modified positions with respect to the original sequence. Another peptide, YHVYKKVNTLLCKT (pep9), reported a performance similar to the original peptide, despite being highly modified by six substitutions in both the core and flanking regions. We highlight that these two peptides were identified within a relatively small pool of 17 peptides. In addition, the binding remains stable for these peptides (**Figure 2E**) after 24 hours, which is crucial for MHC presentation (43). From a virtual screening perspective, this is a highly positive result that motivates us to design epitopes for multiple-allele binding.

### 2.3 Multiple-Allele Binding Engineering of a *P.vivax* Epitope

We selected the epitope from *P. vivax* with sequence DYDVVYLKPLAGMYK, which has been assayed against multiple MHC II alleles, and with positive immunological responses in an animal model (44). An advantage of this epitope is that it belongs to the Merozoite Surface Protein (MSP-1), which is also used as a source of epitopes for *P. falciparum* (45) Because of the available experimental data, and the differences in binding affinities between the MHC II alleles: DRB1*01:01 (*IC*_50_= 1 nM), DRB1*15:01 (*IC*_50_ = 792.9 nM), DRB1*04:01 (*IC*_50_ = 1636.1 nM) and DRB1*03:01 (*IC*_50_ = 17807.9 nM), the sequence is a suitable starting point for the design of a better multiple-allele epitope. Note that this epitope is quite active toward allele DRB1*01:01, but the affinity is several orders of magnitude smaller for the other alleles, being worst for allele DRB1*03:01.

**Figure 3:**
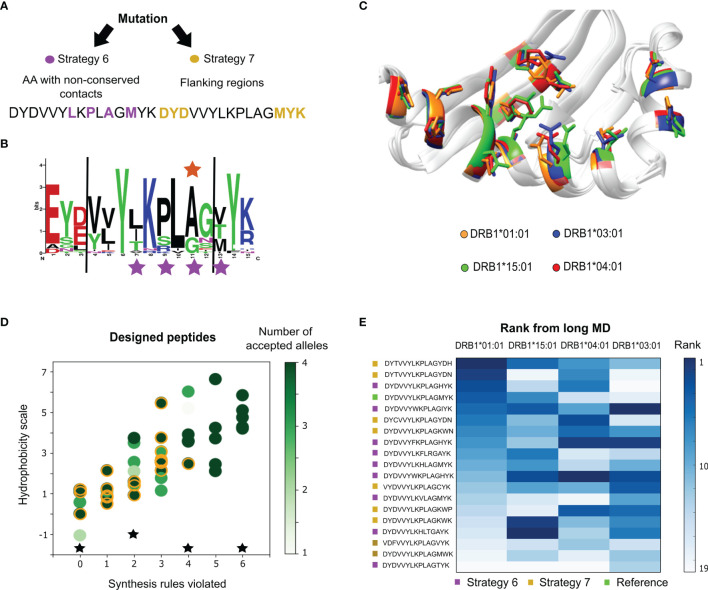
*P. vivax* epitope design for multiple-allele binding. **(A)** Mutation strategies implemented by randomly modifying amino acids in the core region with low conservation (strategy 6), and those belonging to the peptide flanking regions (strategy 7). **(B)** Logo representation of the alignment showing the frequency of the epitope amino acids at each position. The positions modified during design strategy 1 are marked with purple stars, and the reported polymorphism is marked with an orange star. **(C)** Structural view of the polymorphic amino acids from the MHC II β-chain. **(D)** Hydrophobicity values, the number of synthesis rules violated and the number of alleles where the consensus score criterion was improved for the designed peptides. From a total of 42 peptides, 18 were selected for long MD simulations (orange circles). The controls are represented as black stars. **(E)** Average rank of the peptide candidates for the four alleles estimated from the long MD. The darker the shade of blue, the higher the rank. The peptides are color-coded according to the two mutation strategies from panel **(A)** The reference peptide is labeled green.

Two alternative mutation protocols were attempted (**Figure 3A**) using the multiple-allele engineering presented in section 2.1 (**Figure 1B**). For the first mutation strategy (strategy 6 in **Figure 3A**), we selected peptide positions that were in contact with non-conserved amino acids of the alleles as evinced by a multiple sequence alignment of orthologs of the MSP-1 antigen in *Plasmodium* species (positions 7, 9, 11 and 13, see **Figure 3C**). The motivation for this mutation protocol is that mutations in contact with polymorphic residues of the target might impact binding, without dramatically disrupting the immune response. The second mutation strategy (strategy 7 in **Figure 3A**) consisted of modifying amino acids that belong to the flanking regions. This allows the conservation of core interactions, and avoids drastically affecting immunogenicity. In this protocol the design was run by modifying any of the 6 flanking amino acids randomly.

The design for multiple-allele binding was run for both strategies, obtaining 42 designed sequences. Most of the substitutions are found in the C-terminal region, obtaining sequences with a maximum of 4 substitutions from the original peptide. In **Figure 3D**, the number of alleles with improved scores is shown as a function of the hydrophobicity scale and the number of empirical-synthesis rules violated per peptide. To select a small set of candidates, we also took into account the number of empirical solubility rules violated (see Methods). From these results, a total of 18 designed sequences (yellow circles in **Figure 3D**) were prioritized and subjected to MD simulations of 100 ns (see **Supplementary Tables 7, 8**). The long MD was used to calculate an average rank per allele using the individual rank of each scoring function evaluated over the last half of the trajectory (**Figure 3E**). We note that most sequences predicted better affinities for the alleles with the worst experimental *IC*_50_ values (*i.e.*, DRB1*04:01 and DRB1*03:01), which is ideal for balancing binding among the four alleles. We also subjected four known epitopes to the same scoring protocol: the PADRE epitope (AKFVAAWTLKAAA), an influenza immunogenic epitope (PKYVKQNTLKLAT), the Vimentin peptide (SAVRLRSSVPGVR) and the natural CLIP substrate (PVSKMRMATPLLMQA), which were used as controls. According to the average scores, the controls (for most cases) ranked better than the reference epitope (see **Supplementary Figure 2**). With respect to the designed peptides, the controls are ranked in intermediate positions.

**Figure 4:**
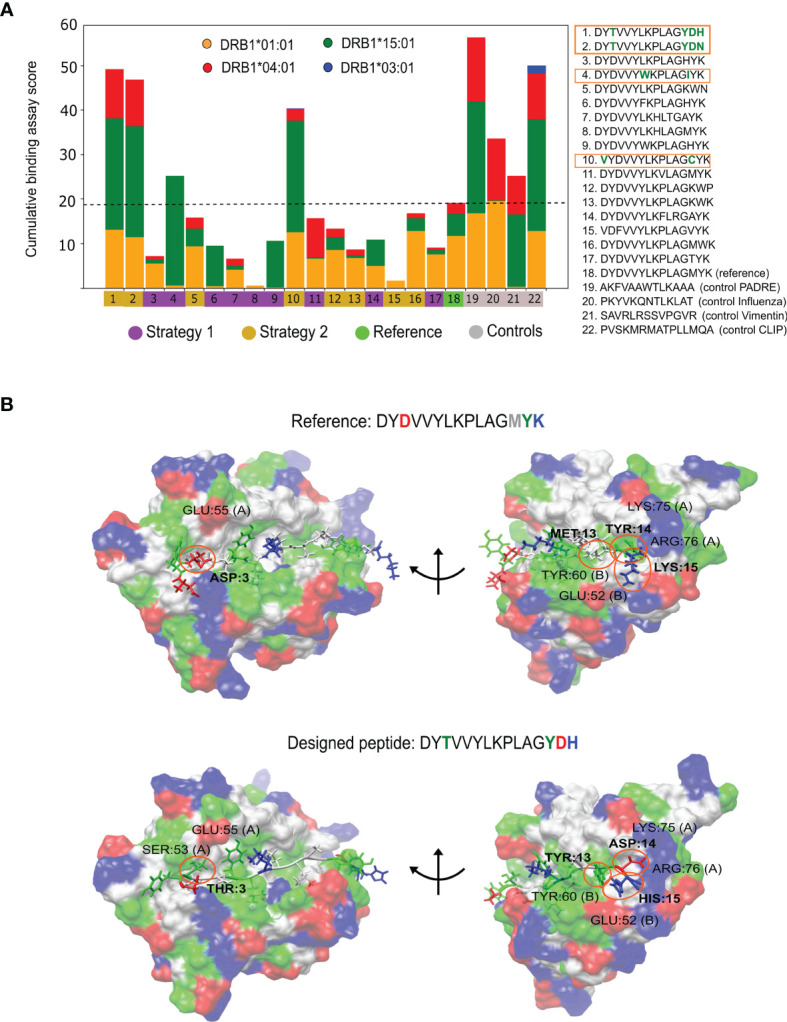
Experimental findings for the *P. vivax* engineered peptides. **(A)** Cumulative Multiple-allele REVEAL score for the selected peptides and the controls toward the four alleles included in the study. The human MHC II (HLA) alleles are represented as: DRB1*01:01 in orange, DRB1*15:01 in green, DRB1*04:01 in red and DRB1*03:01 in blue. The peptides with the best experimental results are enclosed in orange. The black dash line is the activity achieved by the reference peptide, values above it indicate a better performance. **(B)** Bound conformation of best designed peptide (bottom) and the reference peptide (top) to the DRB1:01*01 allele. Representative structures are taken from the long MD simulations. The mutated amino acids are shown in bold in the sequences, and labeled on the structures to highlight their physical interactions. Acidic amino acids are colored in red, basic in blue, polar in green and non-polar in white.

The 18 designed candidates and the additional controls were synthesized and analysed using the ProImmune REVEAL^®^ MHC-peptide binding assay to determine their level of binding to the MHC II alleles DRB1*01:01, DRB1*03:01, DRB1*04:01 and DRB1*15:01. A multiple-allele score was measured to evaluate if the peptides improved toward multiple alleles (**Figure 4A**). Of the 18 peptides, the peptide DYCVVYLKPLAGYDN could not be synthesized by the Prospector PEPscreen^®^ technology. This peptide has the highest hydrophobicity scale (highest yellow circle in **Figure 3D**), indicating that the pre-selection filters are important. From the remaining set, we found that four of the peptides bound better than the majority of the alleles with respect to the reference epitope. None of the peptides, including the controls, were able to trigger a signal for the allele DRB1*03:01 (see **Figure 4A**). This is probably because the affinity of the original reference is too low toward this allele (four orders of magnitude in comparison to DRB1*01:01) and the peptides’ signal is not resolvable with respect to the internal control of the ProImmune experiment. Therefore, we cannot draw conclusions if the designed peptides are better or worse than the reference for this allele.

Three out of the four peptides with better binding affinities were obtained from the strategy where only flanking amino acids were modified. These results suggest that the flanking region is prone to mutations that successfully increase the multiple-allele affinity. The ProImmune REVEAL^®^ scores were also internally weighted based on the potential impact of the peptides in the population (see **Supplementary Figure 3**). Remarkably, we observed that one of the peptides (DYTVVYLKPLAGYDH) is superior to the PADRE epitope toward the studied alleles, which is a major achievement of this study.

Using long MD simulations, we investigated the physicochemical interactions that stabilize the designed peptides. In **Figure 4B**, we compared the interactions of the reference epitope to those created by the best-performing designed peptide using allele DRB1*01:01. These peptides differ by four mutations: a D3T in the C-terminal, and M13Y, Y14D and K15H in the N-terminal regions. Note that the charge distributions are quite different: -2 charge at the C-terminal and +1 at the N-terminal of the reference in comparison to -1 and 0, respectively, for the best-designed peptide. These differences have a significant impact on binding. The D3T avoids unfavorable negative-negative charge interactions with residue GLU:55 chain A (left side **Figure 4B**). The M13Y, Y14D and K15H mutations present a perfect complement to the charge and hydrophobic distribution of the receptor (right side **Figure 4B**). We highlight the Y14D replacement where the aspartic acid makes tight salt-bridges with residues LYS:75 or ARG:76 (see **Supplementary Note 3** for an analysis of the other designed peptides).

We note that our method is the first to engineer modifications on MHC II epitopes to design neoepitopes for multiple-allele binding. So far, machine learning state-of-the-art methods have been focused on scoring pre-existing sequences (18, 46). We investigated how the ranks predicted with these methods compare with the experimental rank (extracted from the multiple-allele REVEAL^®^ score) of the 18 *P. vivax* designed peptides. In **Supplementary Figure 4A**, we present the predicted ranks for NetMHCIIPan 4.0 (23), the IEDB consensus tool (19), and MARIA (25) toward the resolved experimental alleles. Although some of these methods rank a couple of the peptides favorably, their scores are in general poorly correlated to the experiments, and some of the best peptides are not recognized as such. Considering the four best-performing designed peptides as hits, we calculated an enrichment plot: number of hits as a function of the best-ranked peptides for each method (see **Supplementary Figure 4B**). We find that our method PanMHC-PARCE has the highest enrichment.

### 2.4 *Ex vivo* Experiments

In order to verify if the peptides are able to induce a cellular immune response *ex vivo*, C57BL/6 mice were immunized with individual peptides in the presence of AddaVax adjuvant, while the control group received only the adjuvant (see **Supplementary Figure 5A**). Fifteen days after the last dose, splenocytes from immunized mice were incubated with each peptide to evaluate the number of specific IFNγ-producing cells by ELISpot. For *P. vivax* engineered peptides (see **Supplementary Figure 5B**), we observed that splenocytes from mice immunized with DYDVVYWKPLAGIYK and its reference epitope DYDVVYLKPLAGMYK, presented the highest numbers of specific IFNγ-producing cells (228 and 295 SFU/10^6^ cells, respectively) when compared to groups immunized with peptides DYTVVYLKPLAGYDH, DYTVVYLKPLAGYDN or VYDVVYLKPLAGCYK (127, 54 and 145 SFU/10^6^ cells, respectively). Remarkably, the *P. vivax* designed peptides also presented cross-reactivity, suggesting that the stimulation across this set of peptides is transferable (see **Supplementary Figure 6**).

The engineered Influenza peptide also retained its ability to induce a specific immune response (see **Supplementary Figure 5C**), since YSKYKKQNTLKAAT induced a similar number of specific IFNγ-producing cells compared with the reference epitope YPKYVKQNTLKLAT (473 and 463 SFU/10^6^ cells, respectively). Furthermore, Influenza peptides induced a similar IFNγ response when compared with the previously described PADRE epitope (AKFVAAWTLKAAA). In contrast, the control group showed a negligible specific response. Regarding the controls, we used the Influenza peptide (YPKYVKQNTLKLAT) for the groups that received the different *Plasmodium* peptides in the immunization. On the other hand, the PADRE peptide was used as a negative control for the groups immunized with the Influenza peptides. Our results showed that there was zero response using these controls. Thus, we were able to observe that the production of interferon-gamma was specific, since no significant interferon-gamma production was observed using uncorrelated peptides (see **Supplementary Table 9**).

We note that the designed peptides were not optimized for the mouse H-2-IAb allele, which differs by at least 10 mutations in the binding pocket with respect to the human alleles assessed above (see **Supplementary Figure 7**). Even if the peptides were not designed for that allele, their quality can be assessed by following the same procedure described in Methods. In particular, we ran long MD simulations of the best *P. vivax* and Influenza peptides toward the mouse MHC allele and ranked them using the same sampling/scoring approach. Our computational predictions of the rank of these peptides to the H-2-IAb allele correlate relatively well with the *ex vivo* experimental rank (see **Supplementary Table 10** for *P. vivax* and **Supplementary Table 11** for the Influenza design). Together, these results demonstrate that improving the binding of peptides to MHC molecules can conserve the induction of cellular immune responses, even for alleles which are significantly different from the human alleles.

## 3 Discussion

In this work, we have introduced a computational design protocol to improve the affinity of epitopes toward human MHC II alleles. The key novelty is that the method enables the engineering of peptides with immunological properties for many alleles simultaneously. A first validation performed on a single allele indicated which and how many positions on the peptide can be modified in order to improve peptide-binding. These findings were used to guide a multiple-allele binding design of a reported *P. vivax* T-helper epitope. The *in vitro* experiments proved that four designed peptides had a multiple-allelic performance superior to the reference *P. vivax* epitope. Out of the mutation strategies explored, and for the particular case of multiple-allele optimization, we found that the most promising results are obtained with the mutation strategy that modified the flanking regions (strategy 7 in **Figure 3A**), finding 3 of the 4 best performing peptides, including the best. An additional advantage of this strategy is that by modifying the flanking regions, there is a reduced risk of drastically changing the interaction with the T-cell receptor and the immune response pathway.

It was previously demonstrated that the use of binding prediction algorithms allows for the identification of T-cell epitopes that could be used for epitope-driven vaccine design (47). Our contribution may lead to a better choice of epitopes capable of inducing immune responses as well as binding to multiple alleles, covering the majority of the target population (48). Here, we observed cross-species recognition of T-cell epitopes since they were able to bind tit*in vitro* to human MHC II (DRB1*01:01, DRB1*15:01, DRB1*04:01 and DRB1*03:01) and also elicit *ex vivo* T-cell responses in H-2b mice. Indeed, this phenomenon has previously been described for natural T-cell epitopes from several pathogen-derived antigens (49–51). For example, the *P. vivax* epitope DYDVVYLKPLAGMYK was able to induce a strong immune response in mice and non-human primates (44). Here, we have shown that C57BL/6 mice immunized with two doses of four engineered peptides based on the DYDVVYLKPLAGMYK epitope presented a potent and specific cellular immune response. In addition, we observed that the predicted DYDVVYWKPLAGIYK peptide induced an IFNγ response similar to that observed in the reference peptide. T-cell immunity is also considered vital for the containment of the spread of influenza infection and for minimizing the period of illness, since antibodies generated after vaccination are not able to keep up with the frequent antigenic drift (52). The modifications introduced in the YPKYVKQNTLKLAT peptide showed an improvement in its ability to bind to human MHC II, as well as in its ability to induce similar production of IFNγ when compared to the reference peptide. Therefore, epitopes were able not only to bind to MHC molecules, but also to induce strong T-cell immunity.

Regarding the protocol, the combination of a consensus strategy based on six scoring functions and molecular dynamics sampling is an efficient alternative to exhaustive computational methods that calculate more accurate but time-consuming observables, such as binding free energies (53). This is necessary given the large number of mutations that must be explored in order to find optimal candidates, as well as to guarantee the convergence of the protocol. In this sense, the better the sampling of the peptide sequence space, the higher the probability of detecting peptides with good experimental properties. The protocol has been optimized for the MHC II system, which is an experimentally well-characterized receptor bound to many substrates (54). The code is fully open source and publicly available to the design of peptide-binders for different targets (not only MHC II) (42). The protocol is sufficiently flexible that additional criteria, such as peptide stability [*e.g.*, for increasing TCR response (55)], could be included in the protocol. Regarding the computational time, the cost is directly proportional to the total MD simulation time, which scales linearly with the number of alleles. Our calculations for the multiple-allele design (4 alleles) for the *P. vivax* epitope were around 1 microsecond in total. Therefore, if one wants to perform the same number of mutations, the computational cost would be 250 nanoseconds times the number of alleles. We also remark that our approach is “embarrassingly parallel” with respect to the number of alleles, as it is based on performing independent MD simulations. If the user wants to include more alleles, a protocol to model peptides bound to MHC II crystal structures is available in ref (29).

The essence of the design protocol is to exploit structural information and biophysical simulations for exploring, in an unsupervised manner, the peptide sequence and protein-peptide conformations for multiple alleles simultaneously. Machine learning methods have been very successful in identifying whether a predetermined sequence is a potential epitope. These methods have limitations in predicting affinity differences (29), and they are highly dependent on the training set. The results presented in **Supplementary Figure 4** highlight that data-based approaches are less accurate than our approach in predicting novel epitopes. Importantly, our protocol is a genuine design scheme, which allowed us to explore novel sequences that otherwise would not have been available, especially those with random changes in the flanking regions of the peptide. However, in the future, for efficiency purposes it might be useful to combine both data-driven and *ab initio* strategies. A possible direction for future research would be to exploit the efficiency of these tools in a first massive-screening stage, and then use this structural-dynamics protocol for refinement of a smaller set of candidates selected from the previous stage (prior to experimental validation).

Our strategy enables researchers to engineer epitopes for a pathogen of interest, increasing the actionable range of potential antigenic subunits, toward MHC II alleles. In fact, this approach can be generalized to design peptidic adjuvants that can increase the expected cellular response by targeting both MHC II and T-cell receptors simultaneously. Of course the designed peptides must be tested to avoid side-effects associated with molecular mimicry, which are commonly associated with autoimmune events (56). Currently, developing a new vaccine is an expensive, time-consuming and non-trivial process (more so for neglected diseases that lack investment). We consider the multiple-allelic design of great potential in the first steps of epitope optimization for a cheaper and more effective vaccine development.

## 4 Materials and Methods

### 4.1 Multiple Allele Engineering Protocol

In the following section, we present a brief description of the multiple-allele protocol (shown in **Figure 1B**). To begin with an equilibrated system, the starting complexes are sampled with MD simulations for 100 ns (for details about the MD see the section 4.5). After the initial MD sampling, we generated single-point mutations in the peptide sequence following different strategies for each system. The prediction of rotamers for the mutated amino acids was done with Scwrl4 (57). The program was selected based on a previous assessment of single-point mutation protocols (58). After generating the mutation for all complexes, we performed the following minimization and equilibraton procedure in parallel. First, minimization of the predicted side chain alone is performed with Rosetta (38). In order to relocate overlapping atoms and avoid clashes, a second minimization with Gromacs version 5.1.4 (59) is run with the new amino acid and the water molecules surrounding it within a 2 Å radius. A minimization of the full system is performed with a subsequent NVT equilibration of 100 picoseconds (ps). Finally for each mutation, we performed 5 ns of MD simulations to sample the bound conformations. To compute the score, a snapshot of the trajectory was saved every 100 ps for each complex. This sampling/scoring strategy was previously benchmarked using different MHC II-peptide complexes to define the optimal simulation parameters (54).

The conformations are scored with six scoring functions used for protein-protein and protein-peptide affinity predictions: Haddock (60), Vina (61), a combination of DFIRE and GOAP (DFIRE-GOAP) (62, 63), Pisa (64), FireDock (65), and the BMF-BLUUES scoring combination (66, 67). Details of each scoring function are available in ref (54). For the *P. vivax* application, we exchanged BMF-BLUUES for IRAD (68), which correlated better with the experimental affinities of the *P. vivax* epitope to the four alleles (see **Supplementary Figure 8**). Similarly as in ref (69). an average score over all the conformations for each scoring function was used. If the score difference between the previous and current mutation is negative, then the scoring function predicts a good mutation. For each complex, a consensus-by-vote approach is used (see **Supplementary Note 4**), where a mutation is considered favorable if the number of scoring functions that consider it good is three or higher. Finally, the mutation is accepted if the number of alleles that consider it favorable is three of four alleles; otherwise it is rejected. The mutation process is iterated for many attempts.

### 4.2 Influenza Epitope: Optimization Toward a Single MHC II Allele

We first attempted to design peptides with higher affinity toward a single MHC II (HLA) allele. This phase was relevant in identifying key factors, such as how to perform the mutations, which and how many positions can be modified, and how to optimize the parameters used in the protocol.

#### 4.2.1 Starting MHC II-Epitope System

The starting complex for this optimization was the human MHC II (HLA) allele DRB1:01*01 bound to a peptide of 14 amino acids that is part of the Influenza A virus Hemagglutinin antigen (YPKYVKQNTLKLAT). This sequence has a reported bioactivity of *IC*_50_ = 130 nM from a curated dataset of peptide binders against multiple MHC II alleles (20). The starting crystal structure was that of 1DLH (70) from the Protein Data Bank (PDB) (71), which contains a similar peptide that is missing the tyrosine at the N-terminal flanking region. The missing amino acid was modelled using the Rosetta Remodel functionality (72). This complex was first relaxed using Rosetta with the peptide-protein backbone fixed. Then, it was minimized and NVT/NPT equilibrated using Gromacs version 5.1.4 (59). Afterwards, it was subjected to an MD simulation of 100 ns.

#### 4.2.2 Mutation Strategies and Design

To obtain a diverse and optimal set of peptide candidates, we performed five independent peptide design runs using different mutation strategies. These included the random selection of amino acids or prioritization of residues based on bioinformatic filters and probability matrices (see **Figure 2A** and **Supplementary Note 1**). The design protocol was run, and resulted in 105 accepted sequences for all runs. To select the sequences for the long MD, we applied an additional filter based on the similarity between each pair of peptides. This was done to avoid the inclusion of very similar sequences and increase the diversity of the library (see **Supplementary Note 2**). 200 ns of MD were performed for 22 peptide candidates bound to the single allele. Using the average rank from the long MD, a total of 17 designed peptides were selected for the competitive binding assay experiments, together with the reference peptide and two positive controls: PADRE and the original Influenza epitope. This allowed the evaluation of a full batch of 20 peptides required for the assay.

### 4.3 Design Engineering of *P. vivax* Epitope Multiple-Allele MHC II Binding

#### 4.3.1 *P. vivax* Epitope

We searched for *P. vivax*-derived peptides with reported affinities toward multiple alleles and immunological assays available at the IEDB database (19). A mySQL version of the database was downloaded and accessed using 15-mer peptides with reported *IC*_50_ values as queries. From that search, we selected an epitope which is part of the merozoite surface protein 1 (MSP-1) antigen (DYDVVYLKPLAGMYK). The peptide has been tested against four human MHC II (HLA) alleles: DRB1*01:01 (*IC*_50_ = 1 nM), DRB1*03:01 (*IC*_50_ = 17807.9 nM), DRB1*04:01 (*IC*_50_ = 1636.1 nM) and DRB1*15:01 (*IC*_50_ = 792.9 nM) (44). In the same work, immune assays were conducted in mice with positive results.

#### 4.3.2 Natural Variants of the Peptide

Sequences of MSP-1 from different *Plasmodium* species, including *P. vivax*, were obtained from the PlasmoDB database (73). These were used to run a multiple sequence alignment with the Clustal Omega program (74) to search for natural variants. The species in the alignment were clustered based on publicly available phylogenetic trees of the protein, given its role as an antigen for malaria vaccine studies (75). In addition, we conducted a search of polymorphisms reported in the region of the *P. vivax* genome that codes for this protein. The information was obtained from the MalariaGEN project (76). The variants were mapped onto the epitope region, looking for silent or non-synonymous mutations that can provide clues as to which positions of the peptide are more susceptible to modification. A Logo representation of the sequence variants was created using WebLogo3 (77).

#### 4.3.3 Starting Structures and Simulations

The selected *P. vivax* epitope was modelled bound with the four MHC II allele (HLA) structures: DRB1*01:01 (PDB id 1DLH), DRB1*03:01 (PDB id 1A6A), DRB1*04:01 (PDB id 1J8H) and DRB1*15:01 (PDB id 1BX2). We noted that these PDBs have different peptides bound to the receptor. We modelled the new epitope by aligning the core regions and mutating position-by-position based on each template. We applied the NetMHCIIpan-3.1 tool (23) to predict the 9-mer core region of the epitope to be modelled. The mutations were performed using the fixbb package from Rosetta (78). The modelling of additional amino acids in the flanking region was made with the Remodel package from RosettaCommons (72), with side chain relaxation. Each modelled complex was subjected to 100 ns of MD simulations as described below.

#### 4.3.4 Mutation Strategies

We aimed at performing minimal modifications on the peptide with the hope of not interfering with the epitope’s immunological activity. From the single-allele peptide design phase, we found that it is possible to improve the peptide’s activity with a small number of mutations (e.g., ≤4), and both the core and flanking regions provide valuable sites. Therefore, we defined two design strategies for multiple-allele binding enhancement (**Figure 3A**). The first strategy (strategy 6 in **Figure 3A**) consists of mutations performed over natural variants and peptide positions in contact with MHC II polymorphisms. We used the starting MD trajectories to monitor the contacts created between the peptide and polymorphic residues from the MHC II β chain. Using a threshold of 4 Å, we detected amino acids in the core region and in the flanking region interacting with highly polymorphic sites. In addition, we analyzed the multiple sequence alignment of the antigenic region in the *P. vivax* epitope to identify non conserved residues in the peptide. Based on this analysis, we selected four positions, three in the core and one in the flanking region (stars in **Figure 3B**), to mutate during the design protocol. The second strategy (strategy 7 in **Figure 3A**) involves modifying only amino acids from the flanking regions, without changing the identified core of the peptide. For both strategies, the probability of generating the new mutation is uniform (*i.e.*, there are no preferential amino acids).

#### 4.3.5 PanMHC-PARCE Design Details

For the design protocol, we used as starting complexes the last frame from the MD simulations of the original *P. vivax* epitope bound to each of four alleles. We ran 100 attempted mutations for each mutation strategy, obtaining a total of 42 new designed sequences for all runs. We evaluated for how many alleles the affinity is improved in comparison to the reference peptide using the consensus criteria for each individual allele. We also calculated the bioinformatic properties and filters, similar to the single-allele phase (see **Supplementary Note 2**: Peptide selection criteria for *P. vivax*). Using these results, we selected 18 new sequences with the desired properties, and a better predicted rank for multiple alleles. This small set was subjected to long MD simulations of 100 ns for the four alleles. We also ran MD simulations of four additional controls reporting positive binding data toward the MHC II: the PADRE epitope (AKFVAAWTLKAAA), an influenza immunogenic epitope (PKYVKQNTLKLAT), the peptide Vimentin (SAVRLRSSVPGVR) and the natural CLIP substrate (PVSKMRMATPLLMQA). The peptides were modelled onto the MHC II binding sites following the methodology explained in ref (54). The set of designed peptides used in the long MD and the controls were experimentally tested. The code used is publicly available and explained in the **Supplementary Note 5**.

#### 4.3.6 Prediction of Selected Peptides Toward the Mouse MHC Allele

The designed sequences from *P. vivax* and Influenza that were included in the *ex vivo* experiment were subjected to binding predictions toward the mouse MHC II allele H-2-IAb. We used the same MD/scoring approach implemented with the human alleles for the long MD simulations (see above), but using as reference the mouse MHC II crystal structure with PDB id 1r5v.

### 4.4 Peptide-Candidate Selection

For each mutation strategy, we performed 100 attempted-mutations. We monitored the evolution of the scores to verify if these attempts were optimizing (i.e., lowering) their values (such as in **Figure 1D**). To combine the results from the different design runs, we used the scores calculated from the 5 ns MD simulations to obtain an average rank for each accepted peptide. Specifically, all the accepted peptides were ranked using each scoring function, and the average rank over the six functions was used to prioritize those peptides that had potentially higher affinities.

This rank was used together with three bioinformatics filters to select the candidates for long MD simulations. Two filters consisted of empirical rules to account for solubility and synthesis issues associated with the peptides. The solubility and synthesis rules describe violations raised by certain amino acid patterns found in the peptide sequence (https://bioserv.rpbs.univ-paris-diderot.fr/services/SolyPep/index.html) (79). The higher the number of violations, the lower the probability of synthesizing the peptide. The third filter used a hydrophobic score of the peptide from the Eisenberg hydrophobicity scale defined for proteinogenic amino acids (80). The thresholds for each filter and details of the empirical rules are described in **Supplementary Note 2**. After applying the selection criteria, a small set of peptide candidates was subjected to longer MD simulations (see the Methods). The last half of the trajectory was used to calculate the average score for the same six scoring functions used in the design. Using the average of each scoring function, an average rank was calculated and used to re-rank the candidates. This long MD re-ranking was used to select the set of designed peptides for the experiments.

### 4.5 MD Simulations

Each protein-peptide complex was subjected to MD simulations with previous minimization and NVT/NPT equilibration phases. The system was minimized using the steepest descent algorithm, with 50000 steps and a maximum force threshold of 10 kJ/mol/nm. NVT and NPT equilibrations were performed for 100 ps using position restraints on the heavy atoms of the protein to allow for the equilibration of the solvent. Gromacs version 5.1.4 (59) was used to perform the MD simulations. The Amber99SB-ILDN protein force-field (81) and TIP3P water model (82) were used. The protein was solvated with a cubic box of water with a distance of 8Å from the furthest atom of the protein. After solvation, counterions of Na^+^ and *Cl* ^-^ were included in the solvent to make the box neutral. The electrostatic interactions were calculated using the Particle Mesh Ewald (PME) method with 1.0 nm short-range electrostatic and van der Waals cutoffs (83). The equations of motion were solved with the leap-frog integrator (84) using a timestep of 2 femtoseconds (fs). The simulation was run using a modified Berendsen thermostat (85) at 350K temperature-coupling, and the Parrinello-Rahman barostat (86). This was done to allow a fast exploration of the conformational space. To maintain the system at this temperature, all the receptor atoms located at a distance greater than 12 Å from any peptide atom were restrained. The atoms from the receptor located at a distance lower than the threshold remained flexible, as well as the peptide.

### 4.6 Experiments

#### 4.6.1 Rate-Binding Assays

A gold-standard method of rate binding experiments against the MHC II alleles was performed with the Proimmune REVEAL^®^ binding assay. The method uses antibody-labelled peptides that emit a signal if native conformations of the complexes are detected. Consequently, we can verify if a peptide binds to a particular MHC II allele and if the complex remains stable. An illustration of the molecular complex and the emitted signal is shown in **Figure 2D**. Using a control baseline, provided by Proimmune, a score (between 0 an 100) determines a proxy affinity toward the MHC II allele within two time points, one at 0 hours and a second after 24 hours. The peptides were synthesized using the Prospector PEPscreen^®^ technology with high purity standards based on quality controls obtained by MALDI-TOF mass spectrometry (87).

For the single-allele binding optimization phase, 17 peptides selected were assayed together with the controls against the DRB1*01:01 allele. For the multiple-allele engineering phase, 18 P*. vivax* engineered epitopes were evaluated for the MHC II alleles DRB1*01:01, DRB1*03:01, DRB1*04:01 and DRB1*15:01. A multiple-allele score was calculated by averaging the scores of each allele, and by weighting each allele based on the their frequencies in the world population. The calculations were provided by Proimmune. These measures were used to evaluate the binding performance of each peptide toward the four alleles simultaneously.

#### 4.6.2 Peptide Synthesis for *Ex vivo* Experiment

The peptides were synthesized by GenScript USA Inc with more than 75% purity to be tested *ex vivo*: reference *P. vivax* (DYDVVYLKPLAGMYK) and four predicted peptides (DYTVVYLKPLAGYDH, DYTVVYLKPLAGYDN, VYDVVYLKPLAGCYK and DYDVVYWKPLAGIYK); PADRE epitope (AKFVAAWTLKAAA); Influenza reference (PKYVKQNTLKLAT) and one predicted sequence (YSKYKKQNTLKAAT). Peptides were resuspended in DMSO (10 mg/mL) and stored at -20 degree Celsius.

#### 4.6.3 Mice and Immunization

Six- to eight-week-old female C57Bl/6 mice were bred at Centro de Desenvolvimento de Modelos Experimentais para Medicina e Biologia (CEDEME) – UNIFESP. All mice were housed in a temperature-controlled, light-cycled facility at the Division of Immunology- UNIFESP. All experiments using mice in this study were approved by the UNIFESP Institutional Animal Care and Use Committee (IACUC) under protocol number 4615161120, and were in accordance with the recommendations of the Federal Law 11.794 (2008), and the Guide for the Care and Use of Laboratory Animals of the Brazilian National Council of Animal Experimentation (CONCEA). For immunization, mice received two doses, at 2-week intervals, with 50μg of each peptide in the presence of AddaVax adjuvant (1: 1 v/v; *In vivo*gen) in a total volume of 100 μL delivered subcutaneous at the base of the tail.

#### 4.6.4 Splenocyte Isolation

Fifteen days after the last dose, mice were euthanized and spleens were aseptically removed. Single cell suspensions were obtained after red blood cells lysis with ammonium chloride potassium (ACK). Cells were then resuspended in R-10 (RPMI supplemented with 10% fetal bovine serum, 2 mM L-glutamine, 1% v/v vitamin solution, 1 mM sodium pyruvate, 1% v/v non-essential amino acids solution, 1% v/v pen strep, 40 μg/mL of gentamicin and 5×10_5_ M 2-mercaptoethanol (all from Gibco). Cell viability and concentration were estimated using a cell counter (CountessTM Automated Cell Counter, Invitrogen).

#### 4.6.5 T Cell ELISpot Assay

IFNγ-producing cells were assessed using Mouse IFNγ ELISPOT kit (BD Bioscience). The procedure was performed according to the manufacturer’s instructions. Briefly, 96-well plates (MAIPS 4510, Millipore) were coated with IFNγ capture antibody and incubated overnight at 4°C. The plates were washed twice and blocked for 2 hours with R10 at room temperature. Splenocytes were incubated for 18 hours at 37°C in 5% CO_2_ in the presence of each peptide (10μg/mL), Concanavalin A (ConA-2.5 μg/mL; positive control) or R10 (negative control). The plates were washed and incubated with biotinylated anti-mouse IFNγ for 2 hours at room temperature. The plates were then washed and incubated with avidin-HRP for 45 minutes at room temperature in the dark. After extensive washes, the spots were developed with 3-amino-9-ethylcarbazole (AEC) (BD Biosciences) and the number of spots were counted using the AID ELISpot Reader System (Autoimmun Diagnostika GmbH, Germany). The number of IFNγ-producing cells/106 splenocytes was calculated subtracting unstimulated values from stimulated. Statistical significance (p - values) was calculated by One-way ANOVA followed by Tukey honestly significantly different (HSD) *post hoc* test. Statistical analysis and graphical representation were performed using GraphPad Prism version 7.0 software.

## Data Availability Statement

The datasets presented in this study can be found in onlinerepositories and the Supplementary Information. The code repository can be found at: https://hub.docker.com/r/rochoa85/panmhc-parce.

## Ethics Statement

The animal study was reviewed and approved by UNIFESP Institutional Animal Care and Use Committee (IACUC).

## Author Contributions

RO developed the code, implemented the bioinformatics tools, ran the analysis and wrote the manuscript. VASL and DSR performed the *ex vivo* experimental studies and reviewed the manuscript. AL and PC assisted in computational analysis, supported the rate binding experiments and wrote the manuscript. All authors contributed to the article and approved the submitted version.

## Funding

This work and RO and PC have been supported by MinCiencias, University of Antioquia and Ruta N, Colombia, the Max Planck Society, Germany. This research was also supported by the Sao Paulo Research Foundation [FAPESP, grant number 2017/17471-7]. VSL received fellowship from FAPESP and DSR. from CNPq.

## Conflict of Interest

The authors declare that the research was conducted in the absence of any commercial or financial relationships that could be construed as a potential conflict of interest.

## Publisher’s Note

All claims expressed in this article are solely those of the authors and do not necessarily represent those of their affiliated organizations, or those of the publisher, the editors and the reviewers. Any product that may be evaluated in this article, or claim that may be made by its manufacturer, is not guaranteed or endorsed by the publisher.

## References

[1] Skwarczynski M Toth I. Peptide-Based Synthetic Vaccines. Chem Sci (2016) 7:842– 854. doi: 10.1039/C5SC03892H 28791117 PMC5529997

[2] Wieczorek M Abualrous ET Sticht J Álvaro-Benito M Stolzenberg S Noé F. Major Histocompatibility Complex (MHC) Class I and MHC Class II Proteins: Conformational Plasticity in Antigen Presentation. Front Immunol (2017) 8:292. doi: 10.3389/fimmu.2017.00292 28367149 PMC5355494

[3] Xin H Glee P Adams A Mohiuddin F Eberle K. Design of a Mimotope-Peptide Based Double Epitope Vaccine Against Disseminated Candidiasis. Vaccine (2019) 37:2430–8. doi: 10.1016/j.vaccine.2019.03.061 PMC6497404 30930005

[4] Candia M Kratzer B Pickl WF. On Peptides and Altered Peptide Ligands: From Origin, Mode of Action and Design to Clinical Application (Immunotherapy). Int Arch Allergy Immunol (2016) 140:211–33. doi: 10.1159/000448756 PMC7058415 27642756

[5] Ott PA Hu Z Keskin DB Shukla SA Sun J Bozym DJ. An Immunogenic Personal Neoantigen Vaccine for Patients With Melanoma. Nature (2017) 527:217–21. doi: 10.1038/nature22991 PMC5577644 28678778

[6] Keskin DB Anandappa AJ Sun J Tirosh I Mathewson ND Li S. Neoantigen Vaccine Generates Intratumoral T Cell Responses in Phase Ib Glioblastoma Trial. Nature (2019) 565:234–9. doi: 10.1038/s41586-018-0792-9 PMC6546179 30568305

[7] Bezu L Kepp O Cerrato G Pol J Fucikova J Spisek R. Trial Watch: Peptide-Based Vaccines in Anticancer Therapy. Oncoimmunology (2018) 7:e1511506. doi: 10.1080/2162402X.2018.1511506 30524907 PMC6279318

[8] Manning JE Oliveira F Coutinho-Abreu IV Herbert S Meneses C Kamhawi S. Safety and Immunogenicity of a Mosquito Saliva Peptide-Based Vaccine: A Randomised, Placebo-Controlled, Double-Blind, Phase 1 Trial. Lancet (2020) 395:1998–2007. doi: 10.1016/S0140-6736(20)31048-5 32534628 PMC9151349

[9] Malonis RJ Lai JR Vergnolle O. Peptide-Based Vaccines: Current Progress and Future Challenges. Chem Rev (2019) 120:3210–29. doi: 10.1021/acs.chemrev.9b00472 PMC7094793 31804810

[10] Alexander J Sidney J Southwood S Ruppert J Oseroff C Maewal A. Development of High Potency Universal DR-Restricted Helper Epitopes by Modification of High Affinity DR-Blocking Peptides. Immunity (1994) 1:751–61. doi: 10.1016/S1074-7613(94)80017-0 7895164

[11] Fraser CC Altreuter DH Ilyinskii P Pittet L LaMothe RA Keegan M. Generation of a Universal Cd4 Memory T Cell Recall Peptide Effective in Humans, Mice and Non-Human Primates. Vaccine (2014) 32:2896–903. doi: 10.1016/j.vaccine.2014.02.024 24583006

[12] Paul S Dillon MB Arlehamn CSL Huang H Davis MM McKinney DM. A Population Response Analysis Approach to Assign Class Ii Hla-Epitope Restrictions. J Immunol (2015) 194:6164–76. doi: 10.4049/jimmunol.1403074 PMC4458389 25948811

[13] Cong H Mui EJ Witola WH Sidney J Alexander J Sette A. Towards an Immunosense Vaccine to Prevent Toxoplasmosis: Protective Toxoplasma Gondii Epitopes Restricted by Hla-a* 0201. Vaccine (2011) 29:754–62. doi: 10.1016/j.vaccine.2010.11.015 PMC3014376 21095258

[14] Xu Z Chokkalingam N Tello-Ruiz E Walker S Kulp DW Weiner DB. Incorporation of a Novel Cd4+ Helper Epitope Identified From Aquifex Aeolicus Enhances Humoral Responses Induced by Dna and Protein Vaccinations. Iscience (2020) 23:101399. doi: 10.1016/j.isci.2020.101399 32763137 PMC7409978

[15] Hos BJ Tondini E van Kasteren SI Ossendorp F. Approaches to Improve Chemically Defined Synthetic Peptide Vaccines. Front Immunol (2018) 9:884. doi: 10.3389/fimmu.2018.00884 29755468 PMC5932164

[16] Wang P Sidney J Dow C Mothé B Sette A Peters B. A Systematic Assessment of MHC Class II Peptide Binding Predictions and Evaluation of a Consensus Approach. PloS Comput Biol (2008) 4:e1000048. doi: 10.1371/journal.pcbi.1000048 18389056 PMC2267221

[17] Zhao W Sher X. Systematically Benchmarking Peptide-Mhc Binding Predictors: From Synthetic to Naturally Processed Epitopes. PloS Comput Biol (2018) 14:e1006457. doi: 10.1371/journal.pcbi.1006457 30408041 PMC6224037

[18] Jensen KK Andreatta M Marcatili P Buus S Greenbaum JA Yan Z. Improved Methods for Predicting Peptide Binding Affinity to Mhc Class Ii Molecules. Immunology (2018) 154:394–406. doi: 10.1111/imm.12889 29315598 PMC6002223

[19] Vita R Overton JA Greenbaum JA Ponomarenko J Clark JD Cantrell JR. The Immune Epitope Database (IEDB) 3.0. Nucleic Acids Res (2015) 43:D405–12. doi: 10.1093/nar/gku938 PMC4384014 25300482

[20] Wang P Sidney J Kim Y Sette A Lund O Nielsen M. Peptide Binding Predictions for HLA DR, DP and DQ Molecules. BMC Bioinf (2010) 11:568. doi: 10.1186/1471-2105-11-568 PMC2998531 21092157

[21] Nielsen M Lundegaard C Lund O. Prediction of Mhc Class Ii Binding Affinity Using Smm-Align, a Novel Stabilization Matrix Alignment Method. BMC Bioinf (2007) 8:1–12. doi: 10.1186/1471-2105-8-238 PMC1939856 17608956

[22] Sturniolo T Bono E Ding J Raddrizzani L Tuereci O Sahin U. Generation of Tissue-Specific and Promiscuous Hla Ligand Databases Using Dna Microarrays and Virtual Hla Class Ii Matrices. Nat Biotechnol (1999) 17:555–61. doi: 10.1038/9858 10385319

[23] Andreatta M Karosiene E Rasmussen M Stryhn A Buus S Nielsen M. Accurate Pan-Specific Prediction of Peptide-MHC Class II Binding Affinity With Improved Binding Core Identification. Immunogenetics (2015) 67:641–50. doi: 10.1007/s00251-015-0873-y PMC4637192 26416257

[24] Bravi B Tubiana J Cocco S Monasson R Mora T Walczak AM. Rbm-Mhc: A Semi-Supervised Machine-Learning Method for Sample-Specific Prediction of Antigen Presentation by Hla-I Alleles. Cell Syst (2021) 12:195–202. doi: 10.1016/j.cels.2020.11.005 33338400 PMC7895905

[25] Chen B Khodadoust MS Olsson N Wagar LE Fast E Liu CL. Predicting Hla Class Ii Antigen Presentation Through Integrated Deep Learning. Nat Biotechnol (2019) 37:1332–43. doi: 10.1038/s41587-019-0280-2 PMC7075463 31611695

[26] Alvarez B Reynisson B Barra C Buus S Ternette N Connelley T. Nnalign_ma; Mhc Peptidome Deconvolution for Accurate Mhc Binding Motif Characterization and Improved T-Cell Epitope Predictions. Mol Cell Proteomics (2019) 18:2459–77. doi: 10.1074/mcp.TIR119.001658 PMC6885703 31578220

[27] Bordner AJ Mittelmann HD. Multirta: A Simple Yet Reliable Method for Predicting Peptide Binding Affinities for Multiple Class Ii Mhc Allotypes. BMC Bioinf (2010) 11:482. doi: 10.1186/1471-2105-11-482 PMC2957400 20868497

[28] Degoot AM Chirove F Ndifon W. Trans-Allelic Model for Prediction of Peptide: Mhc-Ii Interactions. Front Immunol (2018) 9:1410. doi: 10.3389/fimmu.2018.01410 29988560 PMC6026802

[29] Ochoa R Laskowski RA Thornton JM Cossio P. Impact of Structural Observables From Simulations to Predict the Effect of Single-Point Mutations in Mhc Class Ii Peptide Binders. Front Mol Biosci (2021) 8:124. doi: 10.3389/fmolb.2021.636562 PMC8253603 34222328

[30] Juretić D Vukičević D Petrov D Novković M Bojović V Lučić B. Knowledge-Based Computational Methods for Identifying or Designing Novel, Non-Homologous Antimicrobial Peptides. Eur Biophysics J (2011) 40:371–85. doi: 10.1007/s00249-011-0674-7 21274708

[31] Pillong M Hiss JA Schneider P Lin YC Posselt G Pfeiffer B. Rational Design of Membrane-Pore-Forming Peptides. Small (2017) 13:1701316. doi: 10.1002/smll.201701316 28799716

[32] Gladich I Rodriguez A Hong Enriquez RP Guida F Berti F Laio A. Designing High-Affinity Peptides for Organic Molecules by Explicit Solvent Molecular Dynamics. J Phys Chem B (2015) 119:12963–9. doi: 10.1021/acs.jpcb.5b06227 26398715

[33] Soler MA Medagli B Semrau MS Storici P Bajc G de Marco A. A Consensus Protocol for the *In Silico* Optimisation of Antibody Fragments. Chem Commun (2019) 55:14043–6. doi: 10.1039/C9CC06182G 31690899

[34] Bjorkman PJ Saper M Samraoui B Bennett WS Jt S Wiley D. Structure of the Human Class I Histocompatibility Antigen, Hla-A2. Nature (1987) 329:506–12. doi: 10.1038/329506a0 3309677

[35] Jardetzky TS Brown JH Gorga JC Stern LJ Urban RG Yi C. Three-Dimensional Structure of a Human Class Ii Histocompatibility Molecule Complexed With Superantigen. Nature (1994) 368:711–8. doi: 10.1038/368711a0 8152483

[36] Zaidi N Soban M Chen F Kinkead H Mathew J Yarchoan M. Role of *In Silico* Structural Modeling in Predicting Immunogenic Neoepitopes for Cancer Vaccine Development. JCI Insight (2020) 5(17):e136991. doi: 10.1172/jci.insight.136991 PMC7526456 32879142

[37] Xiao Z Zhang Y Yu R Chen Y Jiang X Wang Z. *In Silico* Design of Mhc Class I High Binding Affinity Peptides Through Motifs Activation Map. BMC Bioinf (2018) 19:516. doi: 10.1186/s12859-018-2517-3 PMC6311935 30598069

[38] Alford RF Leaver-Fay A Jeliazkov JR O’Meara MJ DiMaio FP Park H. The Rosetta All-Atom Energy Function for Macromolecular Modeling and Design. J Chem Theory Comput (2017) 13:3031–48. doi: 10.1021/acs.jctc.7b00125 PMC5717763 28430426

[39] Correia BE Bates JT Loomis RJ Baneyx G Carrico C Jardine JG. Proof of Principle for Epitope-Focused Vaccine Design. Nature (2014) 507:201–6. doi: 10.1038/nature12966 PMC4260937 24499818

[40] King C Garza EN Mazor R Linehan JL Pastan I Pepper M. Removing T-Cell Epitopes With Computational Protein Design. Proc Natl Acad Sci (2014) 111:8577–82. doi: 10.1073/pnas.1321126111 PMC4060723 24843166

[41] Yachnin BJ Mulligan VK Khare SD Bailey-Kellogg C. Mhcepitopeenergy, A Flexible Rosetta-Based Biotherapeutic Deimmunization Platform. J Chem Inf Modeling (2021) 61:2368–82. doi: 10.1021/acs.jcim.1c00056 PMC8225355 33900750

[42] Ochoa R Soler M Laio A Cossio P. Parce: Protocol for Amino Acid Refinement Through Computational Evolution. Comput Phys Commun (2021) 260:107716. doi: 10.1016/j.cpc.2020.107716

[43] Sant AJ Chaves FA Jenks SA Richards KA Menges P Weaver J. The Relationship Between Immunodominance, Dm Editing, and the Kinetic Stability of Mhc Class Ii: Peptide Complexes. Immunol Rev (2005) 207:261–78. doi: 10.1111/j.0105-2896.2005.00307.x 16181342

[44] Rosa DS Iwai LK Tzelepis F Bargieri DY Medeiros MA Soares IS. Immunogenicity of a Recombinant Protein Containing the Plasmodium Vivax Vaccine Candidate MSP119and Two Human CD4+T-Cell Epitopes Administered to Non-Human Primates (Callithrix Jacchus Jacchus). Microbes Infect (2006) 8:2130–7. doi: 10.1016/j.micinf.2006.03.012 16797207

[45] Fowkes FJ Richards JS Simpson JA Beeson JG. The Relationship Between Anti-Merozoite Antibodies and Incidence of Plasmodium Falciparum Malaria: A Systematic Review and Meta-Analysis. PloS Med (2010) 7:e1000218. doi: 10.1371/journal.pmed.1000218 20098724 PMC2808214

[46] Peters B Nielsen M Sette A. T Cell Epitope Predictions. Annu Rev Immunol (2020) 7:123–45. doi: 10.1146/annurev-immunol-082119-124838 PMC10878398 32045313

[47] Rosa DS Ribeiro SP Fonseca SG Almeida RR Santana VC Apostolico J. Multiple Approaches for Increasing the Immunogenicity of an Epitope-Based Anti-Hiv Vaccine. AIDS Res Hum Retroviruses (2015) 31:1077–88. doi: 10.1089/aid.2015.0101 26149745

[48] Borthwick N Ahmed T Ondondo B Hayes P Rose A Ebrahimsa U. Vaccine-Elicited Human T Cells Recognizing Conserved Protein Regions Inhibit Hiv-1. Mol Ther (2014) 22:464–75. doi: 10.1038/mt.2013.248 PMC3911893 24166483

[49] Iwai LK Yoshida M Sidney J Shikanai-Yasuda MA Goldberg AC Juliano MA. *In Silico* Prediction of Peptides Binding to Multiple Hla-Dr Molecules Accurately Identifies Immunodominant Epitopes From Gp43 of Paracoccidioides Brasiliensis Frequently Recognized in Primary Peripheral Blood Mononuclear Cell Responses From Sensitized Individuals. Mol Med (2003) 9:209–19. doi: 10.1007/BF03402131 PMC1430984 15208742

[50] BenMohamed L Bertrand G McNamara CD Gras-Masse H Hammer J Wechsler SL. Identification of Novel Immunodominant Cd4+ Th1-Type T-Cell Peptide Epitopes From Herpes Simplex Virus Glycoprotein D That Confer Protective Immunity. J Virol (2003) 77:9463–73. doi: 10.1128/JVI.77.17.9463-9473.2003 PMC187395 12915561

[51] Rosa DS Ribeiro SP Almeida RR Mairena EC Kalil J Cunha-Neto E. A Recombinant Adenovirus Encoding Multiple Hiv-1 Epitopes Induces Stronger Cd4+ T Cell Responses Than a Dna Vaccine in Mice. J Vaccines Vaccination (2011) 2:1000124. doi: 10.4172/2157-7560.1000124 PMC3693478 23814696

[52] Soema PC van Riet E Kersten G Amorij JP. Development of Cross-Protective Influenza a Vaccines Based on Cellular Responses. Front Immunol (2015) 6:237. doi: 10.3389/fimmu.2015.00237 26029218 PMC4432795

[53] Wan S Knapp B Wright DW Deane CM Coveney PV. Rapid, Precise, and Reproducible Prediction of Peptide-MHC Binding Affinities From Molecular Dynamics That Correlate Well With Experiment. J Chem Theory Comput (2015) 11:3346–56. doi: 10.1021/acs.jctc.5b00179 26575768

[54] Ochoa R Laio A Cossio P. Predicting the Affinity of Peptides to Major Histocompatibility Complex Class II by Scoring Molecular Dynamics Simulations. J Chem Inf Modeling (2019) 59:3464–73. doi: 10.1021/acs.jcim.9b00403 31290667

[55] Rudd BD Brien JD Davenport MP Nikolich-Žugich J. Cutting Edge: Tlr Ligands Increase Tcr Triggering by Slowing Peptide-Mhc Class I Decay Rates. J Immunol (2008) 181:5199–203. doi: 10.4049/jimmunol.181.8.5199 PMC2728084 18832671

[56] Carrasco Pro S Lindestam Arlehamn CS Dhanda SK Carpenter C Lindvall M Faruqi AA. Microbiota Epitope Similarity Either Dampens or Enhances the Immunogenicity of Disease-Associated Antigenic Epitopes. PloS One (2018) 13:e0196551. doi: 10.1371/journal.pone.0196551 29734356 PMC5937769

[57] Peterson LX Kang X Kihara D. Assessment of Protein Side-Chain Conformation Prediction Methods in Different Residue Environments. Proteins: Structure Funct Bioinf (2014) 82:1971–84. doi: 10.1002/prot.24552 PMC5007623 24619909

[58] Ochoa R Soler MA Laio A Cossio P. Assessing the Capability of *in Silico* Mutation Protocols for Predicting the Finite Temperature Conformation of Amino Acids. Phys Chem Chem Phys (2018) 20:25901–9. doi: 10.1039/C8CP03826K 30289133

[59] Hess B Kutzner C van der Spoel D Lindahl E. GROMACS 4: Algorithms for Highly Efficient, Load Balanced, and Scalable Molecular Simulations. J Chem Theory Comput (2008) 4:435–47. doi: 10.1021/ct700301q 26620784

[60] Dominguez C Boelens R Bonvin AMJJ. HADDOCK: A Protein Protein Docking Approach Based on Biochemical or Biophysical Information. J Am Chem Soc (2003) 125:1731–7. doi: 10.1021/ja026939x 12580598

[61] Trott O Olson AJ. AutoDock Vina: Improving the Speed and Accuracy of Docking With a New Scoring Function, Efficient Optimization, and Multithreading. J Comput Chem (2009) 31:455–61. doi: 10.1002/jcc.21334 PMC3041641 19499576

[62] Yang Y Zhou Y. Specific Interactions for Ab Initio Folding of Protein Terminal Regions With Secondary Structures. Proteins: Structure Funct Genet (2008) 72:793–803. doi: 10.1002/prot.21968 18260109

[63] Zhou H Skolnick J. GOAP: A Generalized Orientation-Dependent, All-Atom Statistical Potential for Protein Structure Prediction. Biophys J (2011) 101:2043–52. doi: 10.1016/j.bpj.2011.09.012 PMC3192975 22004759

[64] Krissinel E Henrick K. Inference of Macromolecular Assemblies From Crystalline State. J Mol Biol (2007) 372:774–97. doi: 10.1016/j.jmb.2007.05.022 17681537

[65] Andrusier N Nussinov R Wolfson HJ. FireDock: Fast Interaction Refinement in Molecular Docking. Proteins: Structure Funct Bioinf (2007) 69:139–59. doi: 10.1002/prot.21495 17598144

[66] Berrera M Molinari H Fogolari F. Amino Acid Empirical Contact Energy Definitions for Fold Recognition in the Space of Contact Maps. BMC Bioinf (2003) 4:8. doi: 10.1186/1471-2105-4-8 PMC153506 12689348

[67] Fogolari F Corazza A Yarra V Jalaru A Viglino P Esposito G. Bluues: A Program for the Analysis of the Electrostatic Properties of Proteins Based on Generalized Born Radii. BMC Bioinf (2012) 13:S18. doi: 10.1186/1471-2105-13-S4-S18 PMC3434445 22536964

[68] Vreven T Hwang H Weng Z. Integrating Atom-Based and Residue-Based Scoring Functions for Protein-Protein Docking. Protein Sci (2011) 20:1576–86. doi: 10.1002/pro.687 PMC3190152 21739500

[69] Sarti E Zamuner S Cossio P Laio A Seno F Trovato A. Bachscore. A Tool for Evaluating Efficiently and Reliably the Quality of Large Sets of Protein Structures. Comput Phys Commun (2013) 184:2860–5. doi: 10.1016/j.cpc.2013.07.019

[70] Stern LJ Brown JH Jardetzky TS Gorga JC Urban RG Strominger JL. Crystal Structure of the Human Class Ii Mhc Protein Hla-Dr1 Complexed With an Influenza Virus Peptide. Nature (1994) 368:215–21. doi: 10.1038/368215a0 8145819

[71] Berman HM Westbrook J Feng Z Gilliland G Bhat TN Weissig H. The Protein Data Bank. Nucleic Acids Res (2000) 28:235–42. doi: 10.1093/nar/28.1.235 PMC102472 10592235

[72] Huang PS Ban YEA Richter F Andre I Vernon R Schief WR. RosettaRemodel: A Generalized Framework for Flexible Backbone Protein Design. PloS One (2011) 6:e24109. doi: 10.1371/journal.pone.0024109 21909381 PMC3166072

[73] Aurrecoechea C Brestelli J Brunk BP Dommer J Fischer S Gajria B. Plasmodb: A Functional Genomic Database for Malaria Parasites. Nucleic Acids Res (2009) 37:D539–43. doi: 10.1093/nar/gkn814 PMC2686598 18957442

[74] Sievers F Higgins DG. Clustal Omega. Curr Protoc Bioinf (2014) 48:3–13. doi: 10.1002/0471250953.bi0313s48 25501942

[75] Pacheco MA Cranfield M Cameron K Escalante AA. Malarial Parasite Diversity in Chimpanzees: The Value of Comparative Approaches to Ascertain the Evolution of Plasmodium Falciparum Antigens. Malaria J (2013) 12:328. doi: 10.1186/1475-2875-12-328 PMC3848613 24044371

[76] Pearson RD Amato R Auburn S Miotto O Almagro-Garcia J Amaratunga C. Genomic Analysis of Local Variation and Recent Evolution in Plasmodium Vivax. Nat Genet (2016) 48:959. doi: 10.1038/ng.3599 27348299 PMC4966634

[77] Crooks GE Hon G Chandonia JM Brenner SE. Weblogo: A Sequence Logo Generator. Genome Res (2004) 14:1188–90. doi: 10.1101/gr.849004 PMC419797 15173120

[78] Loffler P Schmitz S Hupfeld E Sterner R Merkl R Hughes M. Rosetta:MSF: A Modular Framework for Multi-State Computational Protein Design. PloS Comput Biol (2017) 13:e1005600. doi: 10.1371/journal.pcbi.1005600 28604768 PMC5484525

[79] Santos GB Ganesan A Emery FS. Oral Administration of Peptide-Based Drugs: Beyond Lipinski’s Rule. ChemMedChem (2016) 11:2245–51. doi: 10.1002/cmdc.201600288 27596610

[80] Eisenberg D Weiss RM Terwilliger TC. The Hydrophobic Moment Detects Periodicity in Protein Hydrophobicity. Proc Natl Acad Sci USA (1984) 81:140–4. doi: 10.1073/pnas.81.1.140 PMC344626 6582470

[81] Lindorff-Larsen K Piana S Palmo K Maragakis P Klepeis JL Dror RO. Improved Side-Chain Torsion Potentials for the Amber Ff99sb Protein Force Field. Proteins: Structure Funct Bioinf (2010) 78:1950–8. doi: 10.1002/prot.22711 PMC2970904 20408171

[82] Jorgensen WL Chandrasekhar J Madura JD Impey RW Klein ML. Comparison of Simple Potential Functions for Simulating Liquid Water. J Chem Phys (1983) 79:926–35. doi: 10.1063/1.445869

[83] Di Pierro M Elber R Leimkuhler B. A Stochastic Algorithm for the Isobaric-Isothermal Ensemble With Ewald Summations for All Long Range Forces. J Chem Theory Comput (2015) 11:5624–37. doi: 10.1021/acs.jctc.5b00648 PMC4890727 26616351

[84] Janežič D Merzel F. An Efficient Symplectic Integration Algorithm for Molecular Dynamics Simulations. J Chem Inf Comput Sci (1995) 35:321–6. doi: 10.1021/ci00024a022

[85] Bussi G Donadio D Parrinello M. Canonical Sampling Through Velocity Rescaling. J Chem Phys (2007) 126:014101. doi: 10.1063/1.2408420 17212484

[86] Parrinello M Rahman A. Crystal Structure and Pair Potentials: A Molecular Dynamics Study. Phys Rev Lett (1980) 45:1196–9. doi: 10.1103/PhysRevLett.45.1196

[87] Dave KA Headlam MJ Wallis TP Gorman JJ. Preparation and Analysis of Proteins and Peptides Using Maldi Tof/Tof Mass Spectrometry. Curr Protoc Protein Sci (2011) 63:16–3. doi: 10.1002/0471140864.ps1613s63 21400691

