# Supplementary material for: Multiple-Allele MHC Class II Epitope Engineering by a Molecular Dynamics-Based Evolution Protocol
Source: Front Immunol. 2022 Apr 27;13:862851. doi: 10.3389/fimmu.2022.862851 (PMC9094701; doi:10.3389/fimmu.2022.862851)
Supplement: Supplementary file 1 [file DataSheet_1.zip › Supplementary Material/Supplementary Information.PDF]

Supplementary Material

1 SUPPLEMENTARY NOTES

1.1 Supplementary Note 1: Design strategies using the Influenza epitope to a single MHC II allele

- **Design strategy 1:** the mutations are performed by choosing uniformly random positions and amino acids. The evolution of the scores for this design strategy is shown in Supplementary Figure S1. The acceptance ratio was around 20-25% and a total of 21 mutations were accepted from the 100 attempts. However, this strategy was predicting sequences with large hydrophobic content and large number of charged amino acids. These factors can affect their synthesis and the evaluation of their activity (Supplementary Table S1).
- **Design strategies 2 and 3:** to avoid the above issues, we used bioinformatic properties as additional filters for the PARCE design, in order to decrease the potential hydrophobicity, as well as increase the chances of being synthesized and soluble during the experimental phases. We perform a random mutation in a random position of the peptide. However, we check if the following criteria for the peptide sequence are satisfied: amino acids maintain the hydrophobic score lower than 3, the number of violations of the synthesis rules is lower than 5, and the number of violations of the solubility rules is lower than 2. If the sequence satisfies these criteria, then we perform the MD and the new sequence is accepted if the consensus is fulfilled. A small group of only 14 accepted sequences is shown in Supplementary Table S2. Because of that, in the design strategy 3 we used the same filters but increasing the threshold criteria values by 1. The sequences accepted are shown in Supplementary Table S3, obtaining a higher number of candidates (23) with reasonable hydrophobic profiles.
- **Design strategies 4 and 5:** For design strategy 4, we used a scoring-matrix to select amino acids with higher probability of being found in the core region of the peptide, a 9-mer region crucial for the peptide activity Bjorkman (2015). In particular, we used an improved scoring-matrix previously created for the MHC II DRB1*01:01 allele Ochoa et al. (2021). The replacement is made on any position of the core, only if the difference between the probability of the old vs new amino acid is greater than zero. If the amino acid that will be replaced is found in the flanking regions, the new side chain is chosen randomly. The list of accepted peptides is shown in Supplementary Table S4 with 23 candidates. For design strategy 5, we implemented the matrix probabilities under a different schema using the cumulative probability defined for all the 20 amino acids. Specifically, at each core position, the cumulative probabilities of all 20 amino acids is represented from 0 to 1 by normalizing the motif matrix. This is used to generate a non-uniform distribution of the amino acids at each position depending on the matrix-motif. The larger the probability of an amino acid to be at a certain position, the larger the chance of being selected by a random number between 0 and 1. Similar to the previous case, if the amino acid is in the flanking regions the new side chain is chosen randomly. Applying this strategy, we found a lower number of just 10 candidates (Supplementary Table S5).

1.2 Supplementary Note 2: Peptide selection criteria

Three filters were applied in addition to the scoring-function consensus criteria. Two consisted on empirical rules to account for solubility and synthesis issues associated to peptides. The rules describe violations raised by certain patterns of amino acids found in the sequence Santos et al. (2016). The

higher the number of violations, the lower the probability to validate the peptides experimentally. The solubility-rules violations are:

- Discard if the number of charged and/or of hydrophobic amino acids exceeds 45%
- Discard if the absolute total peptide charge at pH 7 is more than +1
- Discard if the number of glycine or proline is more than one in the sequence
- Discard if the first or the last amino acid is charged
- Discard if any amino acid represents more than 25% of the total sequence

The synthesis-rules violations are:

- Discard if 2 prolines are consecutive
- Discard if the motifs DG and DP are present in the sequence -2 rules, one per motif
- Discard if the sequences ends with N or Q residues
- Discard if there are charged residues every 5 amino acids
- Discard if there are oxidation-sensitive amino acids (M, C or W) - 3 rules, one per amino acid.

The third filter was the calculation of a peptide-hydrophobic score using the Eisenberg hydrophobicity scale defined for proteinogenic amino acids Eisenberg et al. (1984).

1.2.1 Similarity-criterion filter

To avoid very similar sequences for the final selection of Influenza design peptide set, we used an additional filter to select the most diverse set of sequences. Specifically, an alignment position by position was done, assigning a value to each match/mismatch based on a structure-based position-specific scoring matrix Prlic et al. (2000). The similarity metric between peptide A and B is defined as

$$S_{AB} = \frac{s_{AB}}{\sqrt{s_{AA} s_{BB}}} \,, \tag{S1}$$

here s_{AB} is the alignment score between the two peptides, s_{AA} and s_{BB} are the alignment scores for each peptide with itself. If two peptides report a similarity greater than 80%, only one of the candidates is chosen for the final analysis of the Influenza design.

1.3 Supplementary Note 3: Binding details of P. vivax designed peptides

The second and third best-performing peptides present similar favorable interactions with the receptor (note that the top two only differ by the last amino acid). The fourth best-performing peptide (DYDVVYWKPLAGIYK) was created with the non-conserved amino acid mutation strategy (strategy 6). It differs to the reference by only two mutations: L7W and M13I. The first mutation (L7W) is a position interacting with a buried pocket formed by the two MHC II sub-units, where the tryptophan is able to generate more hydrophobic interactions. In the second mutated position (M13I), the isoleucine favours the generation of more contacts, specially with the polymorphic MHC II residue (Y30) that substantially improves the affinity towards the DRB1*15:01 allele (Figure 4b).

1.4 Supplementary Note 4: Consensus criterion

The mutation is accepted following a consensus-based approach using N scoring functions. If a particular number n of scoring functions agrees on an improvement of the binding affinity of the mutated peptide B, with respect to the one prior to the mutation, *i.e.* peptide A, then the final consensus will accept the

attempted mutation Soler et al. (2019). Formally, the consensus score C is defined as

$$C = \sum_{k=1}^{N} c_k \ , \tag{S2}$$

where c_k for the scoring function k is

$$c_k = \begin{cases} 1, & S_k^B - S_k^A < 0 \ . \\ 0, & otherwise \end{cases} \tag{S3}$$

S_k^I is the value of the average score for peptide I. It should be noted that all employed scoring functions are defined as binding energies, so that lower values means higher binding affinities. The criterion to evaluate if a consensus among the scoring functions is achieved, is based on the comparison of C to a predefined threshold T (with value between 1 and N). If $C \geq T$ the mutated sequence is accepted. The scores are estimated as an average over all the snapshots of the trajectory.

1.5 Supplementary Note 5: PanMHC-PARCE code

PanMHC-PARCE is a docker container to engineer *new* modifications to epitopes bound to different MHC II alleles. The protocol performs a random mutation in the binder sequence, then samples the bound conformations using molecular dynamics simulations in parallel, and evaluates the MHC-peptide interactions from multiple scoring functions. Finally, it accepts or rejects the mutation by applying a consensus criterion based on binding scores and the number of favorable mutations for the majority of alleles (see the Main Text). The procedure is iterated with the aim to explore efficiently novel sequences with potential better affinities toward the specified MHC II alleles. The methodology has been optimized with prepared inputs of multiple MHC II alleles, but it has a design option to use single allele. We provide a tutorial for running and reproducing the methodology. A detailed explanation of the PanMHC-PARCE usage, and tutorial are provided in `https://hub.docker.com/r/rochoa85/panmhc-parce`.

2 SUPPLEMENTARY FIGURES

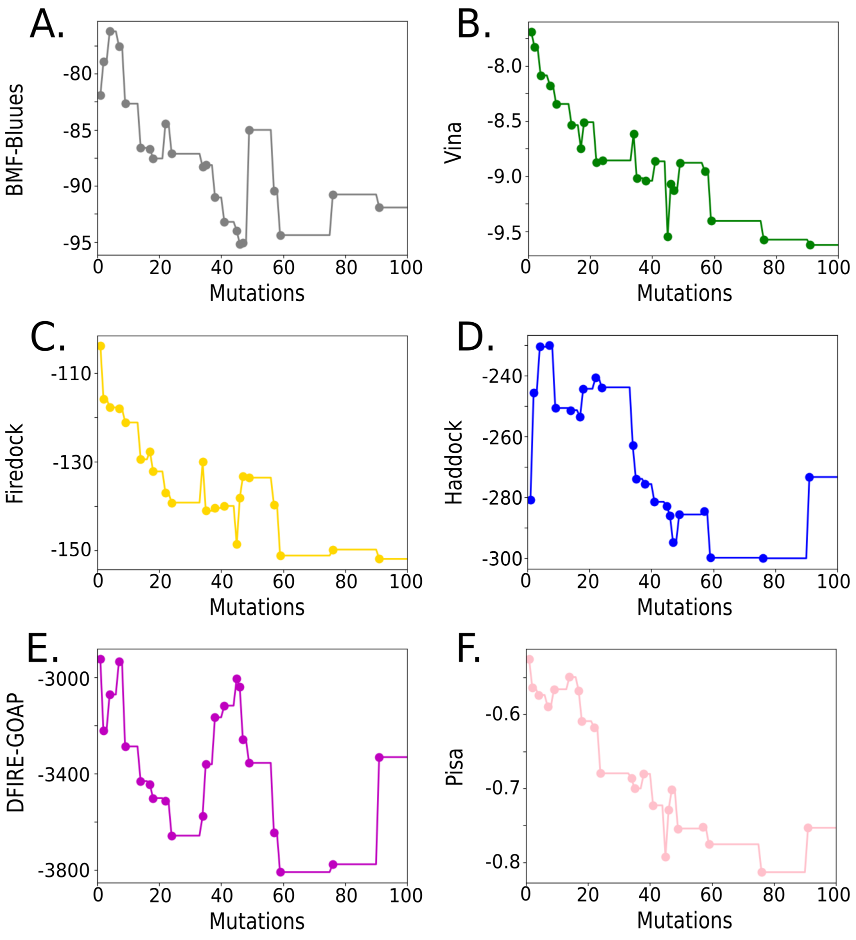

Figure S1. Evolution of the scoring results for design strategy 1 (random mutations and positions). We used six scoring functions to calculate the consensus. The dots in the curve represent the mutations that were accepted. The scoring functions were (A) BMF-Bluues, (B) Vina, (C) Firedock, (D) Haddock, (E) DFIRE-GOAP and (F) Pisa. For the other four strategies, the behaviour of the scoring functions was in general the same as this one.

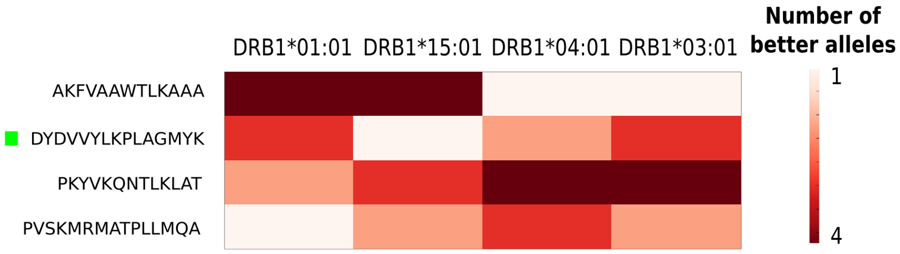

Figure S2. *P. vivax design*: Performance of the control peptides with respect to the reference. We subjected to MD simulations four additional controls having evidence of multiple-allelic performance with the MHC class II alleles assessed in the study. These are the PADRE epitope (AKFVAAWTLKAAA), an influenza immunogenic epitope (PKYVKQNTLKLAT), the peptide Vimentin (SAVRLRSSVPGVR) and the natural CLIP substrate (PVSKMRMATPLLMQA). The number of alleles that are considered better than the reference peptide, according to the average score calculated over the last half of the simulations (last 100 ns), is shown (the darker the higher number of alleles). The reference peptide is labeled with green.

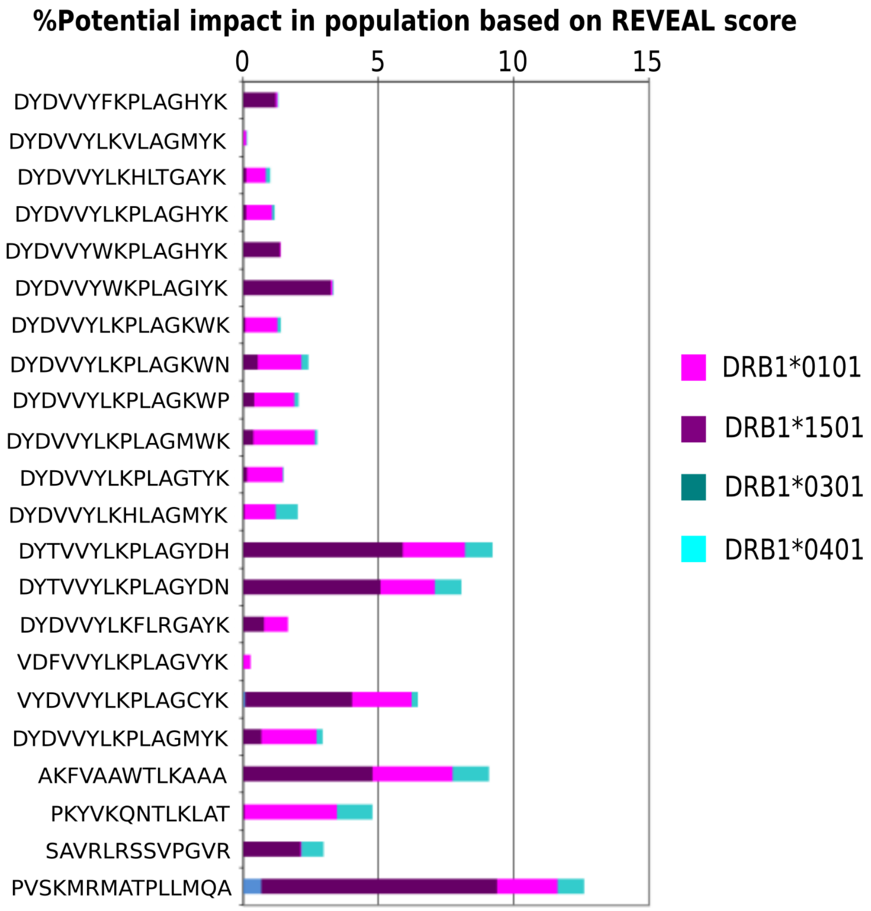

Figure S3. Cumulative Multiple-allele REVEAL® score weighted by the potential impact on the population for the selected peptides and the controls toward the four alleles assessed in the study.

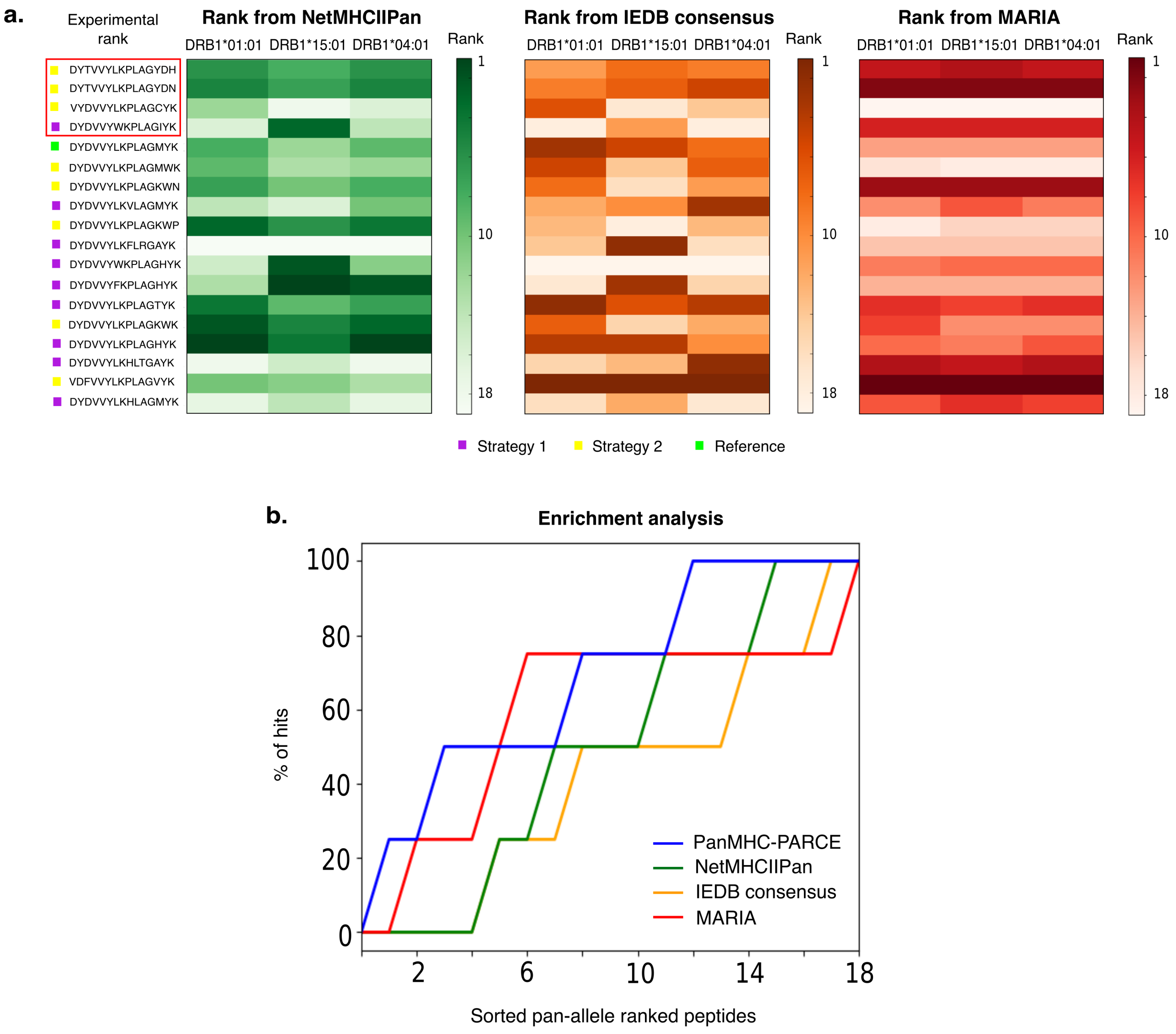

Figure S4. Comparison of state-of-the-art machine learning methods' scoring of the *P. vivax in vitro P. vivax* experimental set. (a) Predicted rank of NetMHCIIPan 4.0 Andreatta et al. (2015), the IEDB consensus tool Vita et al. (2015) and MARIA Chen et al. (2019) towards each allele sorted as a function of the experimental rank. The darker the color, the higher the rank between the set of peptides. The peptides are split into the two design strategies, one using only variable positions of the epitope (purple), and the second using only amino acids from the flanking regions (yellow). The reference peptide is labeled with green. The designed peptides with better experimental performance are highlighted with a red box. (b) Enrichment plot: percentage of hits (designed peptides that are experimentally-scored higher than the reference) as a function of the average predicted-rank (over the alleles) for PanMHC-PARCE, MARIA, NetMHCIIPan 4.0 and IEDB consensus tool. The faster the curve reaches 100%, the higher the enrichment of the scoring prediction.

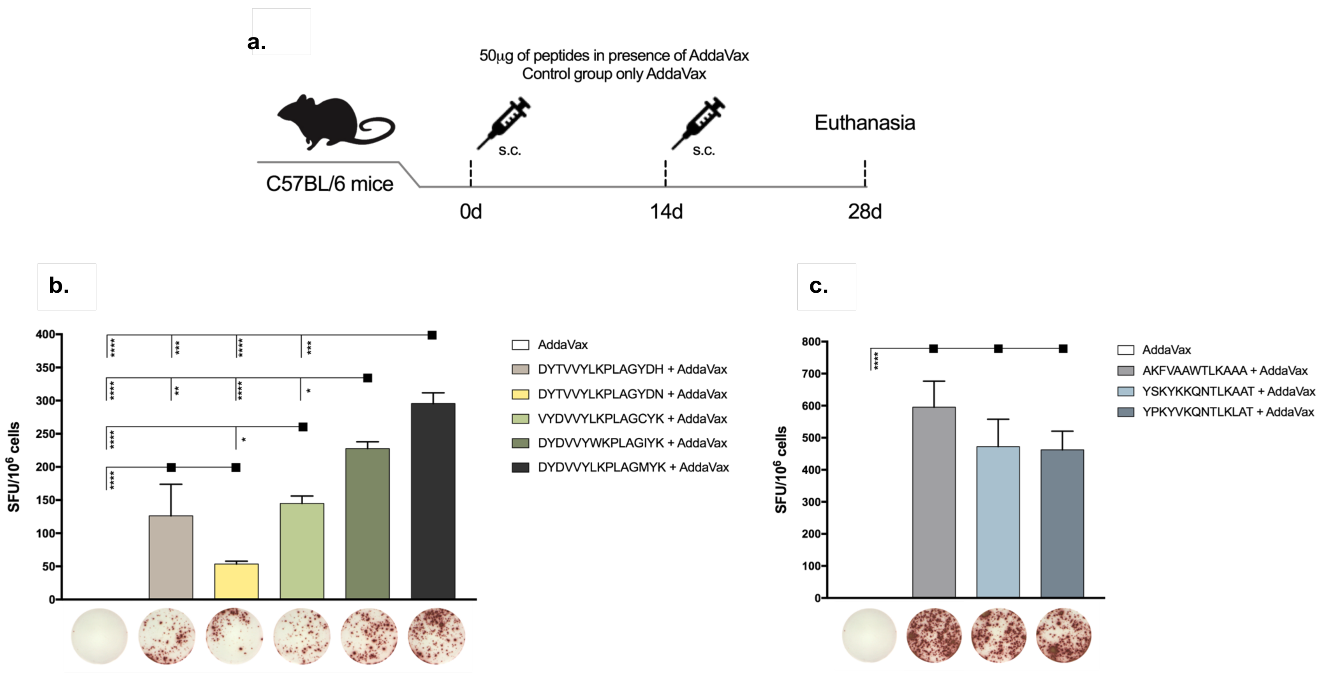

Figure S5. Immune response induced by engineered peptides. (a) Immunization strategy. C57BL/6 mice were immunized s.c. twice with 50 μg of individual peptides in the presence of AddaVax (1:1 v / v). Control groups received only the adjuvant in PBS. (b, c) Fifteen days after the boost, pooled splenocytes were cultured in the presence of 10 mg / mL of each peptide for 18 h to evaluate the number of IFNγ-producing cells by ELISpot assay. Representative wells are shown below the graphs. *p $<$ 0.05, **p $<$ 0.01, ***p $<$ 0.001, ****p $<$ 0.0001. Data represent mean $\pm$ standard deviation (SD). SFU: spot forming units, s.c.: subcutaneous.

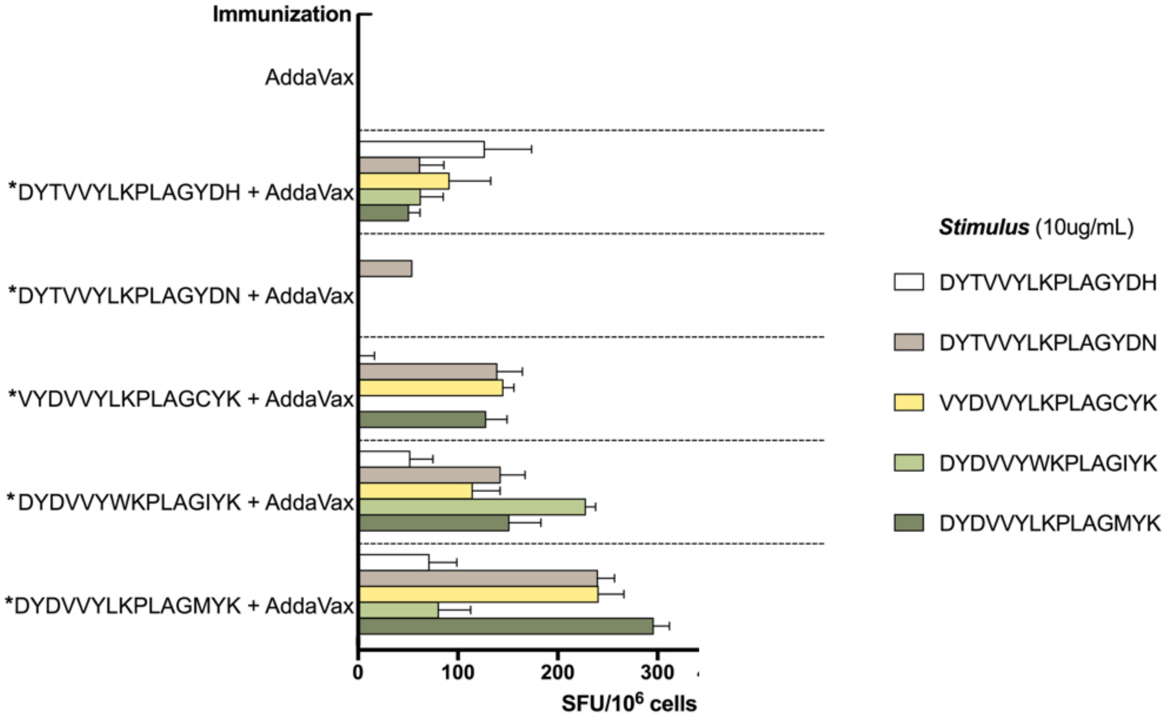

Figure S6. Cross-reactive immune response induced by engineered peptides. C57BL/6 mice were immunized s.c. twice with 50 μg of individual peptides in the presence of AddaVax (1:1 v/v). Control groups received only the adjuvant in PBS. Fifteen days after the boost, pooled splenocytes were cultured in the presence of 10 mg/mL of peptides for 18 h to evaluate the number of IFNγ producing cells by ELISpot assay. These groups were stimulated with the reference peptide (DYDVVYLKPLAGMYK), the four engineered peptides or an uncorrelated peptide as negative control. The control group was stimulated with all peptides. Data represent mean $\pm$ SD (standard deviation). SFU: spot forming units.

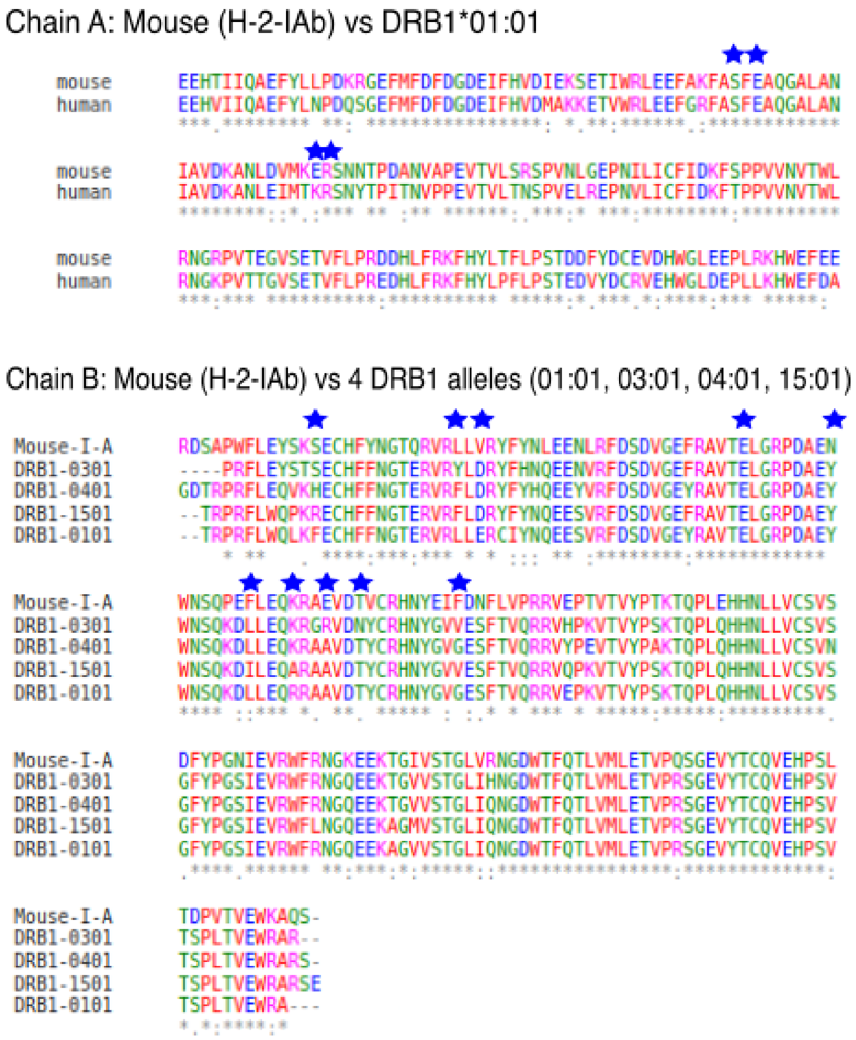

Figure S7. Multiple alignment of the sequences from the mouse H-2-IAb MHC II allele with human alleles. This includes the alignment of mouse H-2-IAb MHC II chain A with HLA chain A from DRB1*01:01 allele, and mouse H-2-IAb MHC II chain B with four HLA chain B alleles (DRB1*01:01, DRB1*03:01, DRB1*04:01 and DRB1*15:01). Positions interacting with the peptides are marked with blue stars.

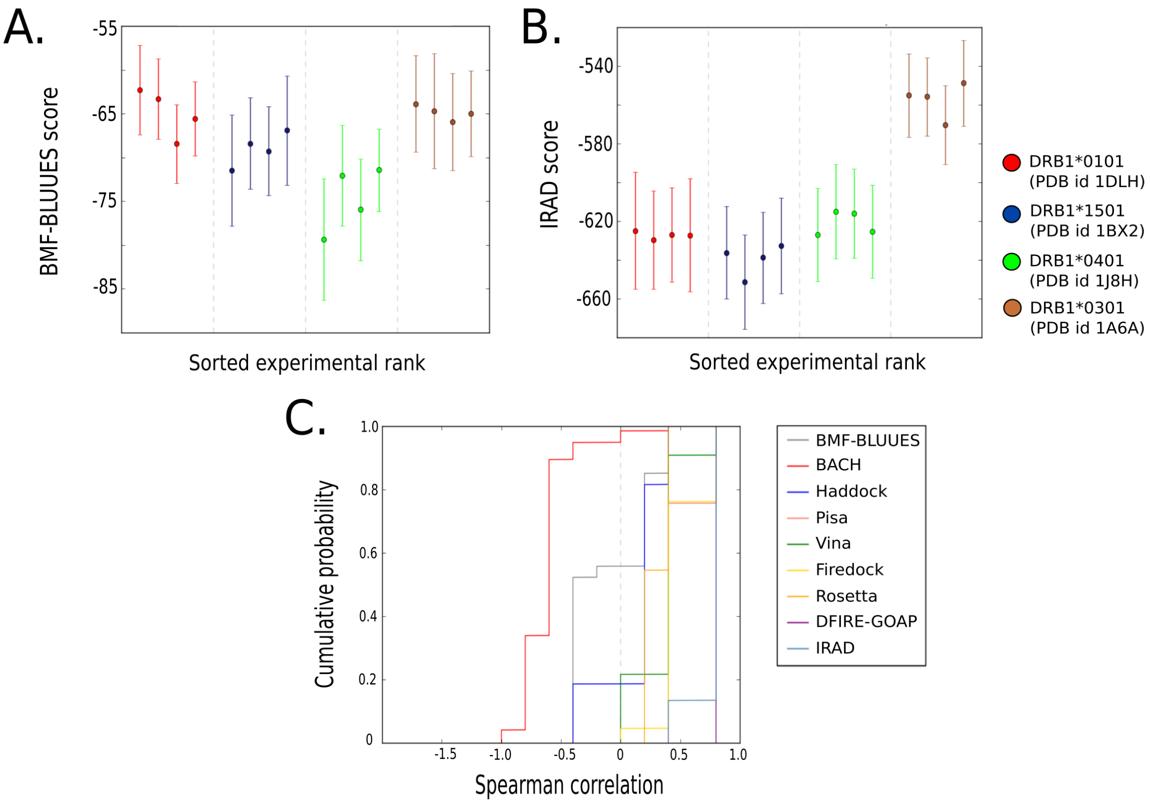

Figure S8. Correlation of the scores for the *P. vivax* reference peptide to the experimental rank of the different alleles. The predictions were calculated from 100 ns of MD simulation using different scoring functions such as (A) BMF-BLUUES and (B) IRAD. This was measured by comparing the experimental sorted rank (given by the affinity to each allele) versus the average score per peptide (split into four blocks). (C) A cumulative probability of the correlation distribution after bootstrapping for 200 replica (similar analysis as in ref. Ochoa et al. (2019)) led us to select BMF-BLUUES instead of IRAD.

3 SUPPLEMENTARY TABLES

Table S1. Peptides selected from the design strategy 1 for the Influenza peptide design, with the mutation performed, the overall hydrophobicity score and the calculation of violations to empirical rules of solubility (Sol.) and synthesis (Syn.).

Sequence	Mutation	Hydrophobicity	Sol. rules violated	Syn. rules violated
YPKYVKYNTLKLAT	QC7Y	-0.66	1	0
YPKYVSYNTLKLAT	KC6S	0.66	1	3
YPKYVSYNTLKVAT	LC12V	0.68	1	3
YPKWVSYNTLKVAT	YC4W	1.23	1	4
YPRWVSYNTLKVAT	KC3R	0.2	1	4
YPRWASYNTLKVAT	VC5A	-0.26	1	4
YVRWASYNTLKVAT	PC2V	0.7	1	4
YVRWAPYNTLKVAT	SC6P	1.01	1	4
YVRWNPYNTLKVAT	AC5N	-0.4	1	4
YVRWNPYNTLKSAT	VC12S	-1.66	1	4
YVRWNPYNTLISAT	KC11I	1.22	0	8
RVRWNPYNTLISAT	YC1R	-1.57	2	8
RVRWNWYNTLISAT	PC6W	-0.88	3	8
RVRWNWYNTWISAT	LC10W	-1.13	3	8
RVRWKWYNTWISAT	NC5K	-1.85	3	6
RVRWKWYNTWSSAT	IC11S	-3.41	3	6
RVRWKWYNVWSSAT	TC9V	-2.28	3	6
RVRWKYYNVWSSAT	WC6Y	-2.83	3	6
RVRWYYYNVWSSAT	KC5Y	-1.07	2	8
RVRWYYYNVWGSAT	SC11G	-0.41	2	8
RVRWYYYCVWGSAT	NC8C	0.66	3	8

Table S2. Peptides selected from the design strategy 2 for the Influenza peptide design, with the mutation performed, the overall hydrophobicity score and the calculation of violations to empirical rules of solubility (Sol.) and synthesis (Syn.).

Sequence	Mutation	Hydrophobicity	Sol. rules violated	Syn. rules violated
YPFYVKQNTLKLAT	KC3F	0.92	1	1
YPFYVHQNTLKLAT	KC6H	2.02	1	1
YPFYWHQNTLKLAT	VC5W	1.75	1	2
YPFYAHQNTLKLAT	WC5A	1.56	1	1
YPFYAHQNTLKLAD	TC14D	0.71	1	1
YPFYAHFNTLKLAD	QC7F	2.75	2	1
YPFYAHFNVLKLAD	TC9V	3.88	2	1
YPFYAHFNFLKLAD	VC9F	3.99	2	1
WPFYAHFNFLKLAD	YC1W	4.54	2	2
WPLYAHFNFLKLAD	FC3L	4.41	2	2
WYLYAHFNFLKLAD	PC2Y	4.55	2	2
WYLFAHFNFLKLAD	YC4F	5.48	2	2
WYLFAHFNFLKQAD	LC12Q	3.57	2	2
WYLFAHFNFLKVAD	QC12V	5.5	2	2

Table S3. Peptides selected from the design strategy 3 for the Influenza peptide design, with the mutation performed, the overall hydrophobicity score and the calculation of violations to empirical rules of solubility (Sol.) and synthesis (Syn.).

Sequence	Mutation	Hydrophobicity	Sol. rules violated	Syn. rules violated
YPKYVKQNVLKLAT	TC9V	-0.64	2	0
YPKYVKQNVYKLAT	LC10Y	-1.44	1	0
YPKYEKQNVYKLAT	VC5E	-3.26	1	0
YPKYAKQNVYKLAT	EC5A	-1.9	1	0
YPKYAKQNVYKLVT	AC13V	-1.44	1	0
YFKYAKQNVYKLVT	PC2F	-0.37	2	0
YFKYAKDNVYKLVT	QC7D	-0.42	2	0
YFKWAKDNVYKLVT	YC4W	0.13	2	1
YFKWAKWNVYKLVT	DC7W	1.84	2	1
YFKWAKWNVYDLVT	KC11D	2.44	1	1
YFKWAKWNVYDLVI	TC14I	3.87	1	1
YFDWAKWNVYDLVI	KC3D	4.47	1	1
YFDWAKWNVYSLVI	DC11S	5.19	1	5
YFDWAKWNVYSHVI	LC12H	3.73	1	2
YFDWAKWNVYSHKI	VC13K	1.15	2	2
YFDWAKWNVYSVKI	HC12V	2.63	1	3
YFMWAKWNVYSVKI	DC3M	4.17	2	4
YFMWLKWNVYSVKI	AC5L	4.61	2	4
YFMWLKWNVYSIKI	VC12I	4.91	2	4
YFMWRKWNVYSIKI	LC5R	1.32	2	3
YFMWRKWNVYPIKI	SC11P	1.62	2	3
YFMWRKWNVYPIKR	IC14R	-2.29	3	3
YFMWRKWNVYPIAR	KC13A	-0.17	3	4

Table S4. Peptides selected from the design strategy 4 for the Influenza peptide design, with the mutation performed, the overall hydrophobicity score and the calculation of violations to empirical rules of solubility (Sol.) and synthesis (Syn.).

Sequence	Mutation	Hydrophobicity	Sol. rules violated	Syn. rules violated
YPKYKKQNTLKLAT	VC5K	-4.35	1	0
YSKYKKQNTLKLAT	PC2S	-4.65	1	0
YSKYKKQNTLKAAT	LC12A	-5.09	1	0
YSKYKKQNTLKCAT	AC12C	-5.42	1	1
YSKYKKQNTLKCKT	AC13K	-7.54	3	1
YSKYKKVNTLKCKT	QC7V	-5.61	3	1
YSVYKKVNTLKCKT	KC3V	-3.03	2	1
YSVYKKVNTLLCKT	KC11L	-0.47	2	3
YHVYKKVNTLLCKT	SC2H	-0.69	2	3
AHVYKKVNTLLCKT	YC1A	-0.33	2	3
AHVYKKLNTLLCKT	VC7L	-0.35	2	3
AHVYKGLNTLLCKT	KC6G	1.63	2	4
AHLYKGLNTLLCKT	VC3L	1.61	2	4
AHLFKGLNTLLCKT	YC4F	2.54	2	4
AILFKGLNTLLCKT	HC2I	4.32	2	4
AILWKGLNTLLCKT	FC4W	3.94	2	4
AILWKSLNTLLCKT	GC6S	3.28	2	4
AYLWKSLNTLLCKT	IC2Y	2.16	2	4
EYLWKSLNTLLCKT	AC1E	0.8	2	4
EVLWKSLNTLLCKT	YC2V	1.62	2	4
HVLWKSLNTLLCKT	EC1H	1.96	3	4
HRLWKSLNTLLCKT	VC2R	-1.65	3	4
HRLWNSLNTLLCKT	KC5N	-0.93	3	7

Table S5. Peptides selected from the design strategy 5 for the Influenza peptide design, with the mutation performed, the overall hydrophobicity score and the calculation of violations to empirical rules of solubility (Sol.) and synthesis (Syn.).

Sequence	Mutation	Hydrophobicity	Sol. rules violated	Syn. rules violated
YPKYVKQNTLKLFT	AC13F	-1.2	2	0
YPKYVKQKTLKLFT	NC8K	-1.92	2	0
YPKFVKQKTLKLFT	YC4F	-0.99	2	0
YPKFVKQKALKLFT	TC9A	-0.32	2	0
YPKFVKQRALKLFT	KC8R	-1.35	2	0
YPKFVTQRALKLFT	KC6T	0.1	2	0
YQKFVTQRALKLFT	PC2Q	-0.87	2	0
YQKFRTQRALKLFT	VC5R	-4.48	2	0
PQKFRTQRALKLFT	YC1P	-4.62	2	0
PQKFRTQRSLKLFT	AC9S	-5.42	2	0

Table S6. Filtered list of peptide candidates for the Influenza design, and ranked after running the MD simulations of 200 ns. The peptides used as Control 1 (PADRE) and Control 2 (Influenza) are also included in the ranking.

Average rank 100 ns MD	Peptide sequence
1	YFMWRKWNVYPIKI
2	YFDWAKWNVYSVKI
3	YFMWRKWNVYSIKI
4	YHVYKKVNTLLCKT
5	EVLWKSLNTLLCKT
6	YPFYAHFNTLKLAD
7	YFDWAKWNVYSHKI
8	PQKFRTQRALKLFT
9	PQKFRTQRSLKLFT
10	YFKWAKWNVYDLVT
11	FKWAKWNVYKLVT
12	AHLFKGLNTLLCKT
13	YPRWASYNTLKVAT
14	YPKWVSYNTLKVAT
15	YQKFVTQRALKLFT
16	YFKYAKDNVYKLVT
PADRE	AKFVAAWTLKAAA
17	YQKFRTQRALKLFT
18	YPFYAHQNTLKLAD
19	YSKYKKQNTLKAAT
Influenza	PKYVKQNTLKLAT
20	YPRWVSYNTLKVAT
21	YPKFVTQRALKLFT
22	YPKYVKQNTLKLAT

Table S7. *P.vivax* design: Selected peptides from the design strategy 6. We include information of the peptide calculated hydrophobicity, the number of violations to solubility and synthesis empirical rules, the number of mutations with respect to the reference peptide, and the number of alleles with better consensus score criterion with respect to the reference. We note that most of the substitutions are associated to positions 9, 11 and 13.

Peptide code	Hydro.	Sol. rules	Syn. rules	Mutations	Number of alleles
DYDVVYLKVLAGMYK	3.08	2	3	1	2
DYDVVYLKPLAGTYK	1.43	3	2	1	1
DYDVVYLKFLRGAYK	0.02	2	0	3	4
DYDVVYLKHLAGMYK	1.60	2	2	1	4
DYDVVYLKHLTGAYK	0.91	2	1	3	4
DYDVVYLKPLAGHYK	1.08	3	0	1	3
DYDVVYFKPLAGHYK	1.21	3	0	2	4
DYDVVYWKPLAGHYK	0.83	3	1	2	4
DYDVVYWKPLAGIYK	2.61	3	3	2	4

Table S8. *P.vivax* design: Selected peptides from the design strategy 7. We include information of the calculated hydrophobicity, the number of violations to solubility and synthesis empirical rules,the number of mutations with respect to the reference peptide, and the number of alleles with better consensus score criterion with respect to the reference.

Peptide code	Hydro.	Sol. rules	Syn. rules	Mutations	Number of alleles
DYDVVYLKPLAGMWK	2.67	3	3	1	2
DYDVVYLKPLAGKWK	0.53	3	1	2	3
DYDVVYLKPLAGKWN	1.25	3	1	3	2
DYDVVYLKPLAGKWP	2.15	3	1	3	3
DYTVVYLKPLAGYDH	2.53	3	3	4	4
DYTVVYLKPLAGYDN	2.15	3	3	4	3
DYCVVYLKPLAGYDN	2.49	3	4	4	4
VYDVVYLKPLAGCYK	3.75	3	3	2	4
VDFVVYLKPLAGVYK	5.47	3	3	4	3

Table S9. Representation of the response against uncorrelated peptides. C57BL/6 mice were immunized s.c. twice with 50 μg of individual peptides in the presence of AddaVax (1:1 v/v). Control groups received only the adjuvant in PBS. Fifteen days after the boost, pooled splenocytes were cultured in the presence of 10 mg/mL of each uncorrelated peptide, as negative control, for 18 hours to evaluate the number of IFN-γ producing cells by ELISpot assay. The number of spots were obtained after subtracting the number of non-stimulated wells.

Groups	Uncorrelated peptides	Number of spots		
AddaVax	all	0	0	0
DYTVVYLKPLAGYDH + AddaVax	YPKYVKQNTLKLAT	0	0	0
DYTVVYLKPLAGYDN + AddaVax	YPKYVKQNTLKLAT	0	0	0
VYDVVYLKPLAGCYK + AddaVax	YPKYVKQNTLKLAT	0	0	0
DYDVVYWKPLAGIYK + AddaVax	YPKYVKQNTLKLAT	0	0	0
DYDVVYLKPLAGMYK + AddaVax	YPKYVKQNTLKLAT	0	0	0
AKFVAAWTLKAAA + AddaVax	YPKYVKQNTLKLAT	0	0	0
YSKYKKQNTLKAAT + AddaVax	AKFVAAWTLKAAA	0	0	0
YPKYVKQNTLKLAT + AddaVax	AKFVAAWTLKAAA	21	0	9

Table S10. Ranking of the *P. vivax* designed sequences towards the mouse MHC allele H-2-IAb used in the *ex vivo* experiments. The computational rank was obtained using MD/scoring approach implemented with the human alleles for the long MD simulations (see the Methods), but using the mouse MHC II allele instead with PDB id 1r5v. The rank from the *ex vivo* experiments are shown in the last column.

Sequence	MD/scoring rank	Experimental rank
DYTVVYLKPLAGYDH (design)	4	4
DYTVVYLKPLAGYDN (design)	5	5
VYDVVYLKPLAGCYK (design)	2	3
DYDVVYWKPLAGIYK (design)	1	2
DYDVVYLKPLAGMYK (reference)	3	1

Table S11. Ranking of the Influenza designed sequences and the PADRE epitope towards the mouse MHC allele H-2-IAb used in the *ex vivo* experiments. The computational rank was obtained using MD/scoring approach implemented with the human alleles for the long MD simulations (see the Methods), but using the mouse MHC II allele instead with PDB id 1r5v. The rank from the *ex vivo* experiments are shown in the last column.

Sequence	MD/scoring rank	Experimental rank
AKFVAAWTLKAAA (PADRE)	1	1
YSKYKKQNTLKAAT (design)	2	2
YPKYVKQNTLKLAT (reference)	3	3

4 SUPPLEMENTARY VIDEOS

4.1 Supplementary Video 1

Animation showing the evolution of the peptide sequences and scoring functions within the PanMHC-PARCE protocol.

REFERENCES

Bjorkman PJ. Not Second Class: The First Class II MHC Crystal Structure. *The Journal of Immunology* **194** (2015) 3–4. doi:10.4049/jimmunol.1402828.

Ochoa R, Laskowski RA, Thornton JM, Cossio P. Impact of structural observables from simulations to predict the effect of single-point mutations in mhc class ii peptide binders. *Frontiers in Molecular Biosciences* **8** (2021) 124.

Santos GB, Ganesan A, Emery FS. Oral Administration of Peptide-Based Drugs: Beyond Lipinski's Rule. *ChemMedChem* **11** (2016) 2245–2251. doi:10.1002/cmdc.201600288.

Eisenberg D, Weiss RM, Terwilliger TC. The hydrophobic moment detects periodicity in protein hydrophobicity. *Proceedings of the National Academy of Sciences of the United States of America* **81** (1984) 140–144. doi:10.1073/pnas.81.1.140.

Prlic A, Domingues FS, Sippl MJ. Structure-derived substitution matrices for alignment of distantly related sequences. *Protein Engineering* **13** (2000) 545–550. doi:10.1093/protein/13.8.545.

Soler MA, Medagli B, Semrau MS, Storici P, Bajc G, de Marco A, et al. A consensus protocol for the in silico optimisation of antibody fragments. *Chemical Communications* **55** (2019) 14043–14046. doi:10.1039/C9CC06182G.

Andreatta M, Karosiene E, Rasmussen M, Stryhn A, Buus S, Nielsen M. Accurate pan-specific prediction of peptide-MHC class II binding affinity with improved binding core identification. *Immunogenetics* **67** (2015) 641–650. doi:10.1007/s00251-015-0873-y.

Vita R, Overton JA, Greenbaum JA, Ponomarenko J, Clark JD, Cantrell JR, et al. The immune epitope database (IEDB) 3.0. *Nucleic Acids Research* **43** (2015) D405–D412. doi:10.1093/nar/gku938.

Chen B, Khodadoust MS, Olsson N, Wagar LE, Fast E, Liu CL, et al. Predicting hla class ii antigen presentation through integrated deep learning. *Nature biotechnology* **37** (2019) 1332–1343.

Ochoa R, Laio A, Cossio P. Predicting the Affinity of Peptides to Major Histocompatibility Complex Class II by Scoring Molecular Dynamics Simulations. *Journal of Chemical Information and Modeling* **59** (2019) 3464–3473. doi:10.1021/acs.jcim.9b00403.
