# Supplementary figures and images for: Multiple-Allele MHC Class II Epitope Engineering by a Molecular Dynamics-Based Evolution Protocol
Source: Front Immunol. 2022 Apr 27;13:862851. doi: 10.3389/fimmu.2022.862851 (PMC9094701; doi:10.3389/fimmu.2022.862851)

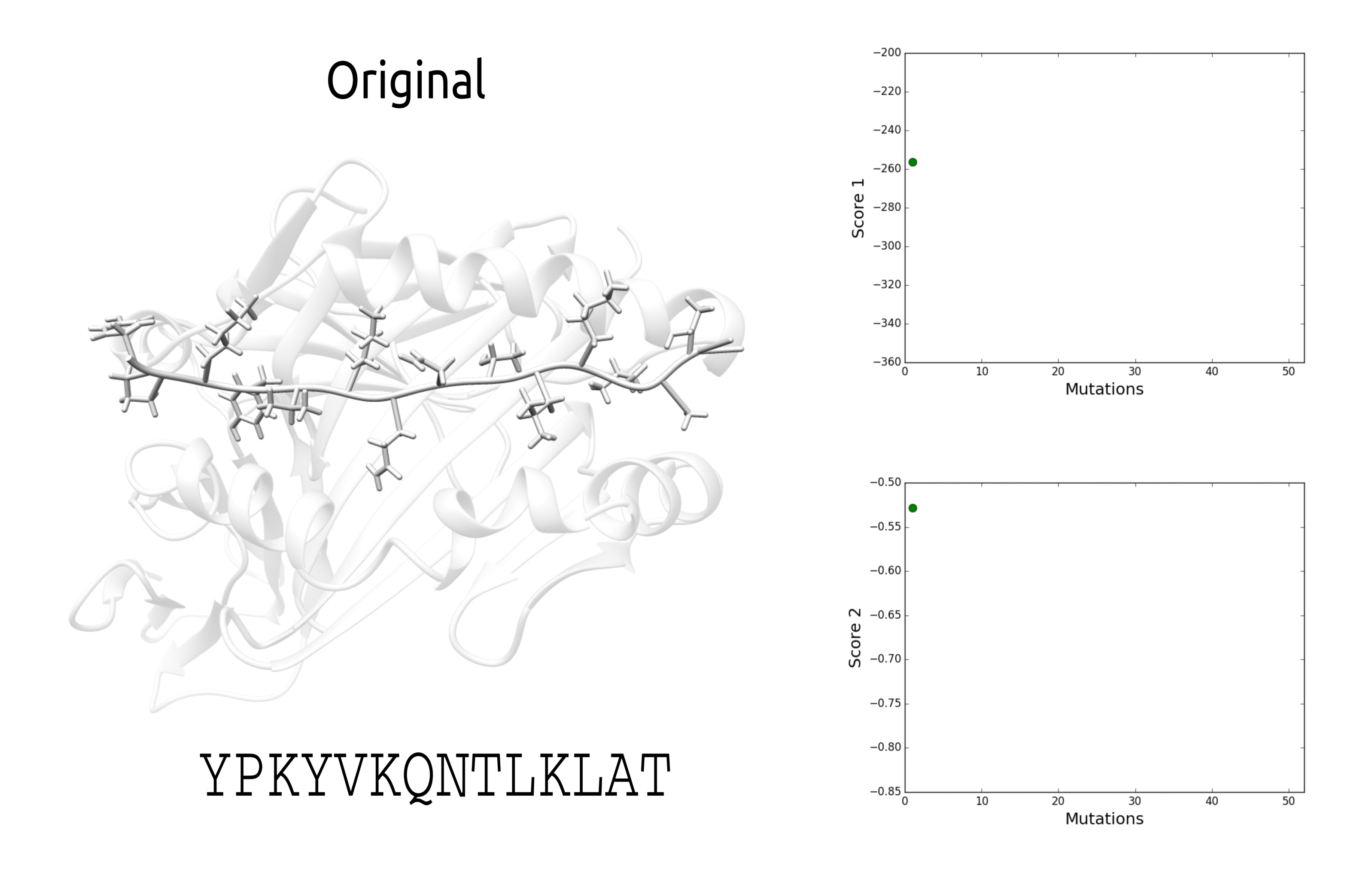

Supplement: Supplementary file 1 [file DataSheet_1.zip › Supplementary Material/Supplementary Video.gif]
